# Stimuli-Responsive Polymeric Nanocarriers for Drug Delivery, Imaging, and Theragnosis

**DOI:** 10.3390/polym12061397

**Published:** 2020-06-22

**Authors:** Sabya Sachi Das, Priyanshu Bharadwaj, Muhammad Bilal, Mahmood Barani, Abbas Rahdar, Pablo Taboada, Simona Bungau, George Z. Kyzas

**Affiliations:** 1 Department of Pharmaceutical Sciences and Technology, Birla Institute of Technology, Mesra, Ranchi, Jharkhand 835215, India; sabya2049@gmail.com; 2 UFR des Sciences de Santé, Université de Bourgogne Franche-Comté, 21000 Dijon, France; priyanshu_bharadwaj@etu.u-bourgogne.fr; 3 School of Life Science and Food Engineering, Huaiyin Institute of Technology, Huaian 223003, China; bilaluaf@hotmail.com; 4 Department of Chemistry, Shahid Bahonar University of Kerman, Kerman 76175-133, Iran; mahmoodbarani7@gmail.com; 5 Department of Physics, University of Zabol, Zabol 98613-35856, Iran; 6 Colloids and Polymers Physics Group, Condensed Matter Physics Area, Particle Physics Department Universidade de Santiago de Compostela, 15782 Santiago de Compostela, Spain; pablo.taboado@usc.es; 7 Health Research Institute of Santiago de Compostela (IDIS), Universidade de Santiago de Compostela, 15782 Santiago de Compostela, Spain; 8 Department of Pharmacy, Faculty of Medicine and Pharmacy, University of Oradea, 410028 Oradea, Romania; sbungau@uoradea.ro; 9 Department of Chemistry, International Hellenic University, 65404 Kavala, Greece

**Keywords:** stimuli-responsive targeting, endogenous stimuli, exogenous stimuli, imaging, drug delivery, stimuli-responsive, theranostic

## Abstract

In the past few decades, polymeric nanocarriers have been recognized as promising tools and have gained attention from researchers for their potential to efficiently deliver bioactive compounds, including drugs, proteins, genes, nucleic acids, etc., in pharmaceutical and biomedical applications. Remarkably, these polymeric nanocarriers could be further modified as stimuli-responsive systems based on the mechanism of triggered release, i.e., response to a specific stimulus, either endogenous (pH, enzymes, temperature, redox values, hypoxia, glucose levels) or exogenous (light, magnetism, ultrasound, electrical pulses) for the effective biodistribution and controlled release of drugs or genes at specific sites. Various nanoparticles (NPs) have been functionalized and used as templates for imaging systems in the form of metallic NPs, dendrimers, polymeric NPs, quantum dots, and liposomes. The use of polymeric nanocarriers for imaging and to deliver active compounds has attracted considerable interest in various cancer therapy fields. So-called smart nanopolymer systems are built to respond to certain stimuli such as temperature, pH, light intensity and wavelength, and electrical, magnetic and ultrasonic fields. Many imaging techniques have been explored including optical imaging, magnetic resonance imaging (MRI), nuclear imaging, ultrasound, photoacoustic imaging (PAI), single photon emission computed tomography (SPECT), and positron emission tomography (PET). This review reports on the most recent developments in imaging methods by analyzing examples of smart nanopolymers that can be imaged using one or more imaging techniques. Unique features, including nontoxicity, water solubility, biocompatibility, and the presence of multiple functional groups, designate polymeric nanocues as attractive nanomedicine candidates. In this context, we summarize various classes of multifunctional, polymeric, nano-sized formulations such as liposomes, micelles, nanogels, and dendrimers.

## 1. Types of Stimuli

It is well established that drugs must be administered in a controlled manner to target sites to boost therapeutic efficacy and reduce or avoid adverse effects. In this approach, stimuli-based drug delivery systems have shown significant potential for the effective targeting of active drug moieties. In 1978, thermosensitive liposomes were first used for drug delivery. Over the years, scientists have designed and extensively used stimuli-responsive biomaterials for controlled drug administration, leading to the birth of the field of stimuli-responsive polymers. Such frameworks have helped in the observation of the physiological causes of illnesses, wherein the proportion of the administered drug is influenced by physiological necessities. There are reports on multiple forms of stimuli, mainly categorized as endostimuli (internal) and exostimuli (external), which have led to effective drug release at targeted sites [1].

### 1.1. Properties of Internal Stimuli 

The intrinsic properties of pathologically challenged tissue differ significantly from healthy normal cells. These properties have helped in designing endostimuli-responsive nanocarriers for the transport and effective targeting of drug cargos.

***pH.*** pH is one of the most commonly used delivery stimuli, employed either on precise organs (vagina/gastrointestinal tract) or on organelles (such as lysosomes, golgi, and endosomes); it has also been used for the release of the drug moieties under altered pathological conditions, like cancer, inflammation, or ischemia with marked pH changes [2]. Extracellular pH is generally maintained at about 7.4 in healthy tissue and blood. Average extracellular pH values are typically acidic due to the high glycolysis rates in many tumors [3]. A low pH can serve to highlight the tumor area to aid site specific drug release. pH-responsive polymers, which are capable of accepting or donating protons in pathological pH, allow moderate conformational changes to occur, and are mostly employed for these systems [4]. Poly(ε-caprolactone) (PCL) nanoparticles have been modified to increase tamoxifen concentrations in estrogen receptor (ER)-positive breast cancer [5]. pH-responsive copolymeric systems can be formed either by introducing an acid functionalized group into the backbone of the polymer which undergoes conformational and solubility variations upon environmental pH change, or by employing acid cleavable bonds that break and permit the release of chemotherapeutics. TNFα was released from a chitosan entity when it was optimized by an amino group upon protonation at the tumor site [6]. Recently, many researches have studied nanoformulations that are derived from natural polysaccharides and modified by pH for successful drug delivery. For example, Chen et al. developed biocompatible cellulose-based hydrogels that were incorporated with pH-sensitive diblock copolymer micelles for prolonged drug delivery [6]. In another study, Luo et al. synthesized amphiphilic stearic acid and carboxymethyl chitosan conjugated self-assembling nanoparticles incorporating paclitaxel. pH stimuli helped in the effective apoptosis of cancer cells via this platform [7]. Saha et al. reported on the development of a pH-triggered auto-fluorescent polymeric nanoplatform for the delivery of nonfluorescent aromatic nitrogen mustard chlorambucil (CBL) to cancer tumors [8]. pH variance is, therefore, a fundamental variable for the evolution of sophisticated DDSs. While pH is widely used in smart drug delivery, it should be coupled with different stimuli, including temperature or redox, to ensure very accurate and precise release at the target sites. The use of acidic pH as a trigger in tumor microenvironments has its limitations. Firstly, acid pH in perivascular regions is usually far away from the blood flow, leading to a lack of nanoparticle response. In addition, pH variations often do not greatly differ in the healthy tissues and tumor tissues [9,10].

***Redοx***. This stimuli system has garnered a lot of attention over the years for the treatment of many ailments, and has been widely explored for intracellular drug delivery systems [10]. The glutathione (GSH) concentration in cancerous tissues is 100–1000 times greater than that in blood, and about 100 times more than that in healthy physiological tissue [11]. GSH, a powerful reducing agent because of its intrinsic thiol group, inhibits reactive oxidative species (ROS) accumulation in diseased tissues and serves as an interesting stimulus for the delivery of anticancer drugs. It has also been shown that the ROS level is 10–100 times greater in inflamed tissue and colon cancer than in normal tissue [12]. Copolymers that effect a response in the presence of glutathione possess disulfide linkage, thereby enabling the formation of micelles which is disrupted in vivo to release the desired drug [13]. Tian et al. fabricated multifunctional mesoporous silica nanocarriers conjugated with transferrin via disulfide linkage to release doxorubicin at cancer sites [14]. Various ROS-reactive DDS forms, including thioether, selenium/tellurium, thioketal, boron ester, peroxalate ester, polyproline, polysaccharide, and aminoacrylates, have been explored in drug administering applications. In a recent study, micelles of PEG_2000_-*S-S*-PTX (PEG conjugated to paclitaxel via disulfide linkage) were manufactured and characterized for use as a redox-sensitive prodrug for breast cancer cells [15]. The redox stimulant DDSs revealed promising sensitivity and precision, but the complex biological climate and heterogenesis nature of cancer cells make it very difficult to achieve the required specificity of the redox reaction.

***Enzymes***. Due to their unique substrate specificity and selectivity, enzyme-responsive DDSs have been extensively studied as an emerging therapeutic field. Many enzymes like lipase, protease, trypsin, glycosidase, phospholipase, oxidoreductase, etc. have been used to aid drug delivery to cancer cells [16,17]. Among the various enzymes, proteases are of supreme interest for the fabrication of novel DDS, since they are often overexpressed in diseases such as cancer and inflammation. Trypsin, a critical digestive proteinase, plays a critical role in regulating the process of exocrine pancreatic secretion, which affects the release of many other digestive enzymes [18]. Radhakrishnan et al. engineered trypsin/hyaluronidase enzyme-triggered hollow nanocarriers to fetch anticancer agents intracellularly [19]. Matrix metalloproteases (MMPs) are a family of endopeptidases that are zinc-dependent; they are famous for their involvement in the prognosis of cancer [20], and have been widely explored for drug delivery as well as in imaging modalities [21]. Zhu et al. fabricated MMP2-sensitive, PEG lipid conjugated liposomes with antinucleosome monoclonal antibodies modified on their surface to enhance cancer targeting [22]. In a different work, Chen et al. fabricated multifunctional poly (ethylene glycol)- blocked-poly(L-lysine) Biotin 6- maleimido- caproic acid (Biotin-PEG-b-PLL(Mal)-peptide) polymeric micelles enclosing doxorubicin to enhance cancer cell uptake by endocytosis [23]. Dendrimer-methoxy poly (ethylene glycol) doxorubicin (DOX) conjugates were also synthesized with the aid of a cathepsin B-cleavable peptide for anticancer targeting, as cathepsin B is overexpressed in tumor microenvironments [24]. Despite its usefulness, enzyme-responsive DDS suffers from a lack of precise control of the initial system response time.

***Hypoxia***. Hypoxia affects tumors in several ways, including angiogenesis, epithelial to mesenchymal transformation, invasiveness and metastasis [25]. Tumor hypoxia represents a promising approach by which to impede tumor growth. Various reducing agents accumulate in hypoxic cells like NADPH, nitoreductase, cytochrome P450 reductase, azoreductase, NADH, and alkaline phosphatase, among others [26]. Myriad modifications caused by hypoxia pose prospective obstacles to the core concepts of nanomedicine architecture. Throughout hypoxic tumor key cells, the hypoxic metabolic cellular pathway can yield lactic acid, making the tumor microenvironment highly acidic. Consequently, many attempts have been made in recent years to develop nanotherapeutics to combat hypoxia, specifically acidic pHs and intracellular redox potential, which trigger anticancer drugs specifically in low oxygen supply tumor cells, but not in healthy, oxygenated cells [27,28]. Several examples of oxygen deprivation-responsive smart DDSs have been established, and have been shown to be useful in the treatment of cancer. Ahmad et al. fabricated hypoxia-responsive, doxorubicin-encapsulating polymeric micelles that demonstrated faster delivery in hypoxic tumor cells [29]. Kulkarni et al. created a hypoxia-specific self-assembly polymersome from polylactic acidazobenzene, a polyethylene glycol that released 90% of the relevant drug in hypoxic tumor conditions [30]. To combat glioma, siRNA anticancer drugs were delivered via hypoxia-sensitive liposomes that exhibited a marked cellular uptake of hypoxic cells [31]. Recently, Yan et al. fabricated gated mesoporous silica nanoparticles that responded to low oxygen concentrations, achieving significant results in both in vitro and in vivo studies [32]. Hypoxia-responsive NPs show restricted extravasation in deep tumor interiors owing to the substantially larger dimensions of synthetic prodrugs. In addition, the drug packing and discharge capacity of NPs is a further constraint to their clinical use.

***Temperature***. Temperature-responsive, smart DDSs have been extensively explored for cancer therapy [33]. The drug release process is governed by a nonlinear abrupt shift in the properties of at least one component regarding the surrounding temperature. Thermosensitive DDSs depend on the speedy delivery of the encapsulated drug when the tumor microenvironment is at an elevated temperature, i.e., about 40–42 °C [2]. For most cases, thermosensitive DDS are liposomes, polymer nanoparticles, or micelles, which are typically made of polymer (N-isopropyl acrylamide). In various several clinical trials, thermoresponsive liposomes (TSLs) have been deemed to be the most specialized thermo-responsive nanodrug delivery system. An increased temperature- or radiofrequency ablation-sensitive doxorubicin liposome, namely ThermoDox^®^ (Celsion Corporation), is in phase II trials, and is intended for use on colorectal liver cancer, hepatocellular carcinoma, and breast cancer [2]. Functional modified thermosensitive liposomes are also under extensive study for specifically targeting the human epidermal growth factor receptor 2 antibody in the treatment of breast cancer [34]. Temperature-sensitive DDS production is typically demanding, and requires the selection of a polymer that is both safe and responsive to minor changes in temperature around normal physiological body temperature (37 °C). Thermosensitive liposomes have reached the advanced stages of clinical trials and are most frequently used.

***Glucose***. Glucose-responsive composites to produce smart insulin DDSs have caught the eye of many researchers. These systems are composed of a glucose-responsive moiety and an insulin vector that detects the blood glucose level [35]. Several modifications of drug carriers like crosslinking, hydrophilicity, and pH are induced in the insulin carrier that regulates the rate of insulin release [36]. Glucose-sensitive hydrogels have been extensively studied over the past few years. Frequently, they utilize immobilized enzymes like glucose oxidase (GOx). These products form with the help of enzymatic reactions in the gel phase transition. A sulfonamide-based, glucose-sensitive hydrogel with glucose oxidase and catalase was fabricated and characterized [37,38]. Glucose-sensitive materials have been formulated by means of carbohydrate-binding proteins, i.e., lectins, as natural receptor-mediated glucose-sensing materials; one of the most widely employed lectins is concanavalin A (ConA) [39]. Various approaches have been studied to understand the conjugation of ConA onto polymers and its encapsulation within microcapsules to aid insulin release in a controlled fashion [40]. Glucose-responsive systems have been diligently studied, and a lot of polymers are available. Jamwal et al. synthesized novel glucose sensitive and in-vitro-pH-responsive insulin DDS from glucose oxidase immobilization on acryloyl cross-linked dextran dialdehyde (ACDD) nanocarriers. The carriers demonstrated the release of 90% of the insulin in artificial intestinal fluid in the presence of glucose [41]. However, it is important to examine the biocompatibility of the delivery materials and their viability for patient use.

In addition to GoX and Con A, boronic acid (BA)-derived polymers have also been the focus of research in the fabrication of glucose-sensitive platforms for drug delivery [42]. Phenylboronic acid (PBA) is among the most widely investigated functional sensing cue for glucose due to its versatile design, high glucose-sensitivity, better stability, and long-term storability compared to protein-based systems (i.e., GOD and Con A) [43]. It can develop reversible covalent complexes with molecules bearing hydroxyl groups. As a Lewis acid, PBA exists in two forms in aqueous solution, i.e., uncharged trigonal BA and charged tetrahedral boronate. The uncharged trigonal PBA-glucose conjugate is unstable at a given pH value, whereas the ionized PBA can firmly bind with glucose moieties [44]. Increasing glucose levels induce the formation of covalent bonds and generate PBA-glucose complexation, which reduces the p*K*_a_ of the trigonal BA. This dynamic equilibrium allows the binding to occur of more glucose to the PBA-functionalized hydrogel, causing the swelling or disassembly of the drug delivery vehicles, resulting in glucose-triggered drug release. The incorporation of PBA into polymers might provide PBA-modified materials with high glucose-sensitivity, making them potential candidates for self-regulated drug delivery [45]. In contrast to natural proteins, BA derivatives possess greater stability and do not induce immune responses in organisms due to their completely synthetic nature. Theoretically, the complexation between glucose and BA produces Donnan osmotic pressure, resulting in a volume phase transition of the hydrogel matrix that will shrink/swell to different extents according to the concentration of glucose [42].

### 1.2. Properties of External Stimuli 

Advancements in medical science have brought about the use of various external stimuli-based energy sources that efficiently trigger drug release from nanocargos for effective delivery to targeted sites. Below, we discuss a few of the stimuli that have used extensively over the past few years.

***Light*.** Light-responsive drug delivery systems, that employ photosensitive carriers, display an on/off drug release mechanism upon irradiation stimulation. For controlled drug delivery, various wavelengths of light (ultraviolet, near infrared, visible) have been reported and discussed. Due to their low penetration, visible as well as UV light were not deemed appropriate for clinical purposes in vivo, whereas the NIR spectrum is considered to be an ideal light source for monitoring drug release due its safety and strengthened tissue penetration [46]. Various mechanisms have been studied for drug release via this system; the first is the photo-thermal effect based on the conversion of light to heat via a photo-thermal agent that disintegrates the nanocapsule to release the drug. Li et al. described multiple nanostructure liposomes loaded with a hydrophilic drug, AMD3100, along with a hydrophobic NIR photo-thermal agent IR780 [47]. Another method which has also recently been used is the two-photon absorption (TPA) method, which relies on the excitation of a molecule from its ground state to a higher energy state with the aid of two photons of equal or different frequencies which are simultaneously absorbed. The technique requires a pulsed laser source with a high energy density to focus on small areas in order to acquire effective, instantaneous energy; this approach has found broad application in biomedical imaging, e.g., the confocal fluorescence microscope of two photons [48]. Following this, photoactivatable micellar systems were fabricated with a copolymeric system containing 2-nitrobenzyl scaffold or 7-diethylamino-4- (hydroxymethyl)coumarin for lipophilic substance drug delivery. Via two-photon NIR irradiation with laser, the prepared micelles disintegrated and distributed the encapsulated drug into aqueous solution [49,50]. Similarly, various drug conjugates have been developed, with a special focus on near infrared.

***Magnetism*.** Due to its freely permeable nature, magnetic stimuli have been employed as a noninvasive method for medical imaging though the process of MRI or for designing controlled drug release platforms [51]. For instance, the most commonly utilized center/shell magnetic nanoparticles (MNPs) display an assortment of unique magnetic traits, and when engineered properly, can give added advantages such as enhanced sites for bioconjugation, enhanced plasma half-life, etc. [52,53]. Two mechanisms are important for controlled drug release in the presence of an external magnetic stimulus: one is hyperthermia [54], and the other is drug targeting guided by a magnetic field [55]. Hypothermia-derived nanocarriers have gained significant attention in recent years. Thirunavukkarasu et al. designed super-paramagnetic iron oxide (Fe_3_O_4_) nanoparticles (SPIONs) and loaded them with SPIONs, along with doxorubicin, in a heat-sensitive PLGA matrix. Upon exposure to a magnetic field, SPIONs generated heat that led to the release of the drug. The success of this platform in both in vivo and in vitro studies revealed its promising chemotherapeutic attributes that should be explored in the future [54]. However, this system needs further investigation to overcome the problem of low drug loading and reduced specificity.

***Ultrasound*.** Due to its many advantages like intrinsic tissue penetration, superior spatiotemporal control, and enhanced safety, ultrasound has been extensively employed as a stimulus in clinical studies Recently, US has been extensively used in clinics as both a diagnostic and therapeutic tool [56]. The use of a technique called “sonoporation” induces temporary or permanent openings in the membranes of the blood vessels, thereby dramatically improving the extravascular transmission of medicinal substances in the area of interest [57,58] due to its intrinsic tissue penetration and safety. Microbubbles are used as the contrast agent for ultrasound. A myriad of vectors were investigated for ultrasonically-facilitated drug delivery, namely polymeric acoustic contrast agents with binding capacity, enhanced lipospheres, as well as nano-/micro- bubble-enhanced therapy [59,60]. For example, Kruskal et al. achieved tumor targeting by nanocarrier- DOX-encapsulated delivery method, followed by ultrasonic tumor irradiation, resulting in the systemic release of the drug [61]. A recent study by Wang et al. designed ultrasound-sensitive oxyl-alkylhydroxylamine (-oa-) linkages between amphiphile segments. Hydrophobic DOX was enveloped between the hydrophobic amphiphile part to aid drug delivery to hepatocellular cancer cells [62].

*Electrical energy*. Applying a weak electrical field to a targeted tissue area following the administration of an electro-sensitive drug can bring about programmed drug delivery via different mechanisms, such as redox reactions [63], carrier structure disruption [64], and through the production of heat from electrical stimulation [65]. Neumann et al. employed local pH variation in electrochemical reactions for controlled drug release. Drug loaded nanofilms were synthesized with a pH-sensitive polymer, i.e., poly (methyl methacrylate-*co*-methacrylic acid) [66]. In a recent work, dextran and aniline trimer-based electrical stimuli-responsive hydrogels were produced for controlled drug release [67].

## 2. Stimuli-Responsive, Polymeric Nanocarriers for Drug and Gene Delivery

### 2.1. Target-Specific Nanocarriers for Efficient Pharmacotherapy

Conventional drug delivery systems (DDSs) have presented severe limitations and challenges which are often due to systemic adverse effects caused by unpredicted bio distribution and the uncontrollable release behavior of drugs. Target-specific nanocarrier systems are among the most typical nanocarrier systems for the delivery of drugs. These systems have helped to overcome the limitations associated with conventional DDSs [68]. Nanocarrier-specific targeted DDSs significantly enhance the therapeutic efficacy of the embedded molecules by precisely targeting them to the diseased cells, tissue, or organs, thereby preventing the embedded moieties from undergoing hepatic first pass metabolism, and thus, enhancing their therapeutic index. Also, these systems have shown significant response and alterations in their properties in the presence of a stimuli (internal or external) [2]. It has been observed that mechanisms at the molecular or cellular levels of drug-loaded nanocarrier systems for targeted delivery play crucial roles, and act simultaneously for effective diagnoses and the management of disease [69]. Although these systems have been extensively used as potential agents in pharmacotherapy, they have exhibited various adverse effects which have limited their clinical and biomedical applications. To attain efficient pharmacotherapy, it is very important for the nanocarrier-DDSs to release the drugs or active moieties selectively at the targeted sites in the body, leading to enhanced therapeutic potential and reduced adverse effects of the enclosed drugs or active moieties. For instance, chemotherapeutic agents which are used for chemotherapy have shown the ability to eradicate the targeted carcinogenic cells; however, due to their cytotoxicity, they also kill normal healthy cells, leading to adverse effects. Nonetheless, nanocarrier-based DDSs have significantly enhanced the therapeutic efficacy, drug residence, and cellular uptake of the incorporated drug or gene at targeted sites with minimum adverse effects [70]. Nanocarriers (NCs) or nanoparticles (NP) could be produced from various organic-based and inorganic-based constituents comprising biodegradable and nondegradable polymers (polymeric nanoparticles (PNPs), polymeric conjugates), lipids (solid-lipid nanoparticles (SLNs), liposomes, and nanoemulsions), dendrimers, micelles, nanocrystals, nanofibers, quantum dots, nanodiamonds, etc. [71]. The literature reveals that the selection of appropriate constituents for the design and production of NCs or NCs-based approaches depends heavily upon the desired pharmacological activity, payload type, disease type, route of administration, and safety profile of the incorporated moieties [69,71]. Stimuli-responsive nanocarrier systems (SNS) have offered a promising platform for the efficient delivery of drugs and genes to targeted sites; such systems act as active contenders, rather than passive carriers. In recent years, lipid- and polymer-based NCs such as PNPs, liposomes, micellar assemblies, dendrimers, and others, have been utilized specifically for the fabrication of SNS. Furthermore, each nanocarrier-based DDS can be efficiently modified to achieve essential stimuli-sensitive activity by either an active or passive targeting approach. SNS has been shown to be among the most significant approaches for the delivery of drugs and genes into targeted sites, as it acts as “trigger” and reacts precisely to biotic stimuli (external or internal) [72,73]. The intra- and extra-cellular pH values of the biological system are significantly affected by disease pathology. For example, in a solid tumor, the extracellular pH is considerably more acidic (∼6.5) than the blood pH (∼7.4) at 37 °C [74]. Additionally, inside the cell, the pH profiles of the lysosomal and endosomal vesicle are substantially lower than those of the cytosol or cytoplasmic matrix. The selection of a proper constituent composition plays a crucial role in the establishment of modified NCs which could efficiently capitalize upon pH variances for the distribution of the incorporated moieties at specific intra or extra cellular sites. Temperature could also be exploited for the release of the nanocarrier-mediated drugs or genes to specific sites [75]. Finally, the concentration level of glutathione (GSH) could be used via disulfide cross-linking with nanocarrier-mediated systems. For instance, these systems have shown substantially enhanced the therapeutic activities, and have improved the targetability of nucleic acid-mediated treatments. Similarly, external stimuli including ultrasonic energy, magnetism, and thermal and light energy have shown potential [76]. For instance, magnetic fields have been extensively used for iron oxide NP targeting. The main mechanism behind such systems is the accumulation of drug-incorporated, magnetic NPs at targeted sites under the influence of an externally guided magnetic field [77]. In the last few year, ultrasound or ultrasonic energy have been used as a potential tool for targeted-DDSs. In some studies, the efficient delivery of active molecules (drugs or biomolecules) at the tumor or cancerous sites has been achieved through local sonication after injecting micellar formulations. This method not only influences the tumor uptake, but also allows for uniform delivery of the drug and micellar assemblies throughout the tumor tissues [78]. Similarly, light-mediated NP-based DDSs have gained considerable attention; light-responsive polymer-based systems which experience inverse interruptions in the presence of light could be an apt means by which to achieve the controlled release of drugs or genes at targeted sites [79].

### 2.2. Active and Passive Targeting

Active and passive approaches have been extensively used to target various NP-based DDSs to achieve efficient systemic therapies. The mechanism of active targeting involves the addition of definite ligands over the NP surface to improve the recognizing ability of cells at the diseased sites. Sometimes, active targeting was achieved by the PEGylation process (addition of PEG), as PEG-modified NCs enhance the circulation time and achieve passive targeting [80]. The endothelial cells in tumors and capillaries expresses particular integrin (IG) receptors (αvβ3 or αvβ5), which could efficiently conjugate with RGD (arginine–glycine–aspartic acid) sequences. RGD-variations have been specifically used to target the NCs into tumors and endothelial cells present over angiogenesis-affected blood vessels. Moreover, any particular peptide sequence could be identified specifically, using the phage display technique [81]. Various studies have reported the use of this method to target recombinant M13 phages [82], rheumatoid arthritis [83], and so on. Recently, a study demonstrated that aptamers and nucleic acid-embedded NCs precisely recognize the prostate tissue antigen over the prostate cancerous cells. Aptamer-associated approaches have provided an additional approach for the active targeting of conjugated NCs into diseased cells [84]. These strategies were found to be more effective when used with specific targeting of the monoclonal bodies which are present at the disease site. For example, HER2 specific antibodies (Trastuzumab^®^ or Herceptin^®^) -altered NPs localized and delivered active moieties explicitly in HER2-overexpressing tumorous cells [85]. Torchilin’s group was able to develop different approaches, including micelles-/liposomes-based systems conjugated with 2C5, a monoclonal antibody which precisely distinguishes antinuclear histones, for the active targeting of drugs to tumor cells [86,87]. In particular, the epidermal growth factor receptors (EGFR) showed overexpressions in prostate or breast cancers; therefore, EGFR acts as a potential candidate for targeting the gene complexes at cancer sites [88]. In addition to tumor or cancer sites, the epithelium of the pulmonary and gastrointestinal tract (GIT) environments, blood vessels lined with endothelial cells, muscular myoblasts, and skin fibroblasts are other potential candidates for targeting gene-based NCs [89]. The NCs not only improved the targetability, but also enhanced the transfection capability by modifying the stimuli-responsive properties. For instance, in one of the studies, pH-triggered PEGylated (lactose attached with PEG) nanogels exhibited endosomolytic capabilities and increased the transfection efficacy [90]. 

Passive targeting is based on the characteristics of the DDSs and the pathological conditions of the disease; it uses these parameters to accumulate and prevent insignificant delivery of the incorporated payloads at specific sites. For example, intravenously administered, PEGylated NCs could specifically accumulate in the tumor microenvironment based on their enhanced permeability and retention (EPR) effect [91]. The EPR effect was also observed in other infections and chronic inflammations; thus, it is expected that NCs or NCs-based systems serve as exhibit therapeutic aids for their treatment [92]. The ability of NCs to distribute in the reticuloendothelial system (RES) also offers hope for the passive targeting of payloads into the macrophages of the spleen and liver. For instance, these approaches could be used for the treatment of infections including leishmaniasis, candidiasis, and listeria [93]. Passive targeting can also be achieved through other approaches, such as specific SNS that distribute bioactive compounds only in the presence of a specific stimulus. For example, compared to drug delivery through polycaprolactone (PCL; non-pH-sensitive polymer) NPs, pH-sensitive poly (beta-amino ester) (PbAE) NPs significantly enhanced drug delivery and targeted tumor sites [72]. Moreover, alterations in the surface charge and size of the NCs could lead to establishment of passive targeting-based approaches. Studies have shown that NCs with a size of less than ~200 nm and bearing positive charge over their surface accumulate and reside in tumor sites for extended periods, compared to neutral or negatively-charged NCs [94]. Kommareddy et al. reported that the passive targeting of gelatin (type B) -based NPs was highly effective in the delivery of genes at tumor sites [95]. In an another study, gelatin (type B) was used for the development of NP-based DDSs incorporating plasmid DNA (pDNA) [96]. The encapsulation of DNA with PEGylated-gelatin NPs exhibited greater efficiency in vitro and in vivo for targeting pDNA-expressed green fluorescent proteins and β-galactosidase [97]. PEGylated-gelatin NPs have also been used to target DNA moieties in the treatment of lung carcinomas and suppressed tumor growth, angiogenesis in breast cancer cells [98]. 

### 2.3. Various Stimuli-Responsive, Polymeric NCs for Drug and Gene Delivery

#### 2.3.1. Internal Stimulus

***pH-responsive*.** The pH of different human body parts varies significantly; from as low as 1.2, it goes up to 7.2. pH-sensitive nano-DDSs are considered very robust for the site-specific delivery of therapeutics to the GIT, as well as to tumor cells, because of the clear demarcation between the tumor intracellular and extracellular pH. These pH-responsive, polymeric DDSs are usually designed using building blocks of polymers that are capable of shifting their charge, and thereby, hydrophilicity, depending on the environmental pH [2]. Two strategies have been employed to devise such stimuli-sensitive, polymeric NCs; the first uses charge-shifting polymers. Polymers which contain a weakly acidic moiety undergo swelling at basic pHs (anionic), while those with alkaline moieties exhibit swelling at acidic pHs (cationic). This leads to basic conformational and/or solubility changes in the polymeric scaffold, that, in turn, leads to drug release [99,100,101,102,103,104]. The second approach is the incorporation of acid-cleavable bonds into the polymeric backbone, resulting in the release of biomolecules [105]. 

***(i) Cationic and Anionic, pH-sensitive polymeric NCs.*** The use of pH-responsive, polymerics for controlled drug delivery started by acknowledging the marked pH variance between the oral mucosa (pH 5.8–7.4) and the stomach (pH 1.0–3.5). In 2002, Hashimoto et al. developed and characterized polyvinylacetal diethylaminoacetate microspheres to masking the taste of trimebutine [106]. More recent studies have been based on understanding the role of the anionic and cationic groups present in the polymeric moiety in drug release. PDPAEMA homopolymer, containing a basic amino group, was used to deliver a poly(ethylene glycol) (PEG)-doxorubicin (DOX) conjugate, after incorporating arginine and histamine groups into the polymer. It was noticeable that at a pH of 5.5, almost 90% of the DOX was released from the scaffold, with a marked cytoxicity against cancer cells [107]. Another study involved nanoparticles made up of 2-(dimethylamino)ethyl methacrylate (DMAEMA) and pyridyl disulfide ethyl methacrylate (PDSEMA). DEAME was complexed with CpG oligonucleotide, while PDSEMA was linked to the antigen, ovalbumin, via disulfide linkage. This polymeric system dissembled at a low endosomal membrane pH to act as a vaccine delivery system [108]. One of the most widely employed amine-containing polymers is chitosan [109]. Chitosan hydrogels have been extensively used to design pH-responsive, polymeric NCs. Chitosan modified with acrylic acid and DMAEMA was formed from the in situ polymerization of free radicals, and helped in delivering 5-fluorouracil, as well as bovine serum albumin. The findings revealed that the volume of 5-FU and BSA released from the gel could be altered by adjusting the formulation makeup, by modulating the concentration of **the** hydrogel as well as the pH of the environment. Toxicity tests verified that empty hydrogels had marginal toxicity to regular cells, while 5-Fu-loaded hydrogels demonstrated sufficient cytotoxicity to LO2 and HepG-2 cancer cells [110]. Recently, novel, biocompatible, carboxymethyl chitosan-layered Pluronic^®^ F68/poly(lactic-co-glycolic acid) (PF/PLGA) nanoparticles were designed and used to deliver a poorly soluble tumor targeting drug, Gefitinib, orally [111]. Poly(N,N-dimethylaminoethyl methacrylate) (PDMAEMA) and poly(N,N-diethylaminoethylmethacrylate) (PDEAEMA) are two more synthetically-derived, cationic, pH-responsive polymers that undergo swelling in acidic conditions because of their intrinsic tertiary amine group protonation [112]. A pH-responsive hydrogel of poly(vinyl alcohol) and PDMAEMA was employed to deliver riboflavin [113]. Many PDEAEMA copolymeric systems have been utilized to deliver anticancer drugs. For example, recently, DOX delivery was achieved using a mixture of polymers, i.e., poly (*N,N*-diethylaminoethyl methacrylate)-*b*-poly(poly(ethylene glycol) methyl ether methacrylate) (PDEAEMA-PPEGMA) with polycaprolactone-*b*-poly (poly(ethylene glycol) methyl ether methacrylate) (PCL-PPEGMA) [114]. Inflamed tissues, just like cancerous cells, possess a unique acidic environment that can be utilized to in the development of targeting scaffolds. Keeping this in mind, by incorporating a polyhistidine moiety to a block copolymeric system of PLGA and PEG, systemic antibiotic delivery was achieved to enhance the uptake of drug at bacterially-infected sites [115].

Anionic systems forming polymers such as albumin, cellulose [116], poly(methacric acid) (PMAA), and poly(aspartic acid) (PAsp), and polymers based on sulfonamide [117] may be used to build nanocarries which become more hydrophobic with a reduction in pH. Functional groups that tend to become hydrophobic in acidic pHs could be used to destabilize the endosomal membranes and help in drug transportation. One work employed poly(propyl acrylic acid) (PPAA) to aid in the intracellular delivery of active pharmaceuticals [118]. The most widely reported anionic polymers include PAA and PMA, along with their derivatives [58]. pH-responsive anionic PAA hydrogels were used to protect the drug from denaturation at low gastric pH [119]. The pH and thermo-responsive copolymeric system of PAA and pNIPAAM (pNIPAAm-b-PAA) was successfully used in DOX delivery [120]. In another study, cationic β-CD-modified chitosan-PAA NCs was fabricated to enhance the drug delivery of paclitaxel (PTX) [121]. Hydrogel made from Arabic gum with glycidyl methacrylate was employed to deliver potassium diclofenac to simulated intestinal fluid [122]. Cationic polymers have also been used for gene delivery. Yu et al. designed amphotericin B-loaded, dual pH-responsive, polymeric micelle-plexes from PDMAEMA diblock copolymers to deliver siRNA [123]. A polymeric pH-responsive hydrogel made from alginate and albumin helped in the delivery of prednisolone [124]. A pH-responsive hydroxyethyl cellulose and hyaluronic acid hydrogel was used to deliver isoliquiritigenin to treat skin lesions and acne [125]. 

***(ii) Acid labile linkers.*** By fabricating NCs which possess covalent pH-responsive bonds that can easily be cleaved at acidic pHs, intrinsic body pH changes can be targeted. Hydrazone linkage is very frequently employed in the design of pH-responsive delivery systems, because the bond is very stable under normal physiological pH conditions, but undergoes hydrolysis in endosomal and/or lysosomal acidic compartments. The first research using hydrazone linkage to deliver drugs was done by Etrych and coworkers, who used *N*-(2-hydroxypropyl)methacrylamide (HPMA) copolymers attached to DOX via hydrazone linkage to achieve controlled drug release in mild acidic conditions [126]. In another study, cisplatin was linked to PEG-b-PLA by hydrazine hydrate. PEG-b-PLA was complexed with the ketone functional groups in Pt(IV) prodrug, and extent of drug release was estimated [127]. In a recent study, galactosyl dextran- retinal nanogels were formulated and attached to alltrans retinal via hydrazone bond [128]. 

Imine bonds have also emerged a successful linker in the design of DDSs. To load melphalan into carboxymethyl cellulose polymeric micelles, hydrogen bonds were used and pyridyl groups were attached to them, facilitating the systemic release of drug [129]. In a recent work, a dual pH- and redox-responsive copolymeric system, formed by the polycondensation of a dimethyl l-cystinate (Cys) and polycaprolactone (PCL) oligomer by imine bond, was used to deliver PTX [130]. Imine-linked dextran- DOX conjugates were also synthesized by oxidizing the hydroxy group of dextran to aldehyde and attaching it to DOX with the help of imine linkage. This system was used to deliver a drug to B16F10 tumors [131].

Acetal bonds undergo pH-dependent hydrolysis to form biocompatible alcohol and aldehydes. A pH-responsive, polymeric micellar DDS was fabricated by acetal-bonded poly(ethylene glycol)-block-polylactide copolymer, and was employed to deliver the anticancer drug PTX. In other research, a novel endosomal pH-responsive PTX prodrug micelle was formed from a poly(ethylene glycol)-poly(ε-caprolactone) (mPEG-PCL) diblock polymer linked via acid-cleavable acetal linkage (mPEG-PCL-Ace-PTX) [132].

The cis-aconityl group of the maleic acid amides (MAA) family was first employed as a pH-stimuli-responsive linkage to fabricate PLL/daunomycin conjugates [133]. DOX was conjugated to PEGylated polyamidoamine (PAMAM) dendrimers via cis-aconityl linkage, as well as acid-insensitive succinic bonds, PPCD and PPSD, respectively [134]. In another study, hyaluronic acid (HA) was functionalized with dual linkage of cis-aconityl and disulfide, fabricating pH and redox dual-responsive, polymeric NCs which released DOX synergistically in the presence of both stimuli, making the self-assembled nanoparticles dual responsive to pH and redox potential [135]. 

***Redox-responsive*.** There is a marked difference in the redox potential between the intracellular and extracellular spaces, the former being highly reductive because of the elevated concentration of glutathione (GSH), and the latter with reductive moieties inside the cell [136]. It has also been demonstrated that tumor tissues are significantly more reducing and hypoxic than normal healthy cells [137]. This ensures the usefulness of the glutathione disulfide-glutathione redox couple (GSSG/GSH) in the development of redox-responsive delivery systems [138]. The most widely exploited redox-responsive linker is the disulfide linker, that can reduce to a thiol group in highly reducing conditions. This results in basic conformational changes in the fabricated delivery system, leading to drug release [139]. The discussion that follows shall divide the topic into two categories: (i) disulfide-linked systems, and (ii) recently studied diselenide linked systems.

***(i) Disulfide-linked systems***. Disulfide linkage can easily be introduced into polymeric systems by groups like L-cysteine, dithiodiglycolic acid, as well as pyridyl disulfide, to develop redox-responsive drug/gene carriers. To counter the problem of nanoparticle stability in vivo, a cross linking technique has been widely employed. The core of the micelle, when cross-linked with a reducible linker, would only break in the reducible environment of the cell. For example, self-assembling, rice-grain shaped, novel redox-responsive, polymeric DDSs were formulated from the diblock copolymeric system of poly(ethylene glycol)-b-poly(lactic acid) (MPEG-SS-PLA) to deliver PTX. In vitro studies revealed that these systems were highly biocompatible, and aided in the endocytosis of PTX [140]. Another example could be of a copolymeric redox-responsive system based on poly(ethylene oxide)-b-poly(N-methacryloyl-N′-(t-butyloxycarbonyl) cystamine) (PEO-b-PMABC) to regulate the release of DOX [141]. Another research group investigated a poly(DTPA-co-Cys) polyionic nanogel to encapsulate curcumin-derived chemotherapeutic agent [142]. To overcome the problem of multidrug resistance, a disulfide-linked block copolymeric redox-responsive micelle of poly(ε-caprolactone), i.e., PCL and poly(ethyl ethylene phosphate) (PEEP), was formulated, i.e., PCL-SS-PEEP, that made it possible to achieve higher accumulations of DOX in MDR cancer cells [143]. In a different study, α-tocopherol succinate, usually used to inhibit drug resistance, was grafted onto hyaluronate amphiphile by disulfide linkage to form a polymeric micelle, and was used to deliver PTX to GSH-expressing cells [144]. DOX- and verapamil-delivering polymeric nanoparticles were made from the block copolymeric system of PEG and a poly(2-methacryloyloxy)ethyl-5-(1,2-dithiolan-3-yl)pentanoate redox-responsive system [145]. A redox-responsive, polymeric micelle incorporating O,N-hydroxyethyl chitosan bridged with octylamine by disulfide bond was developed to deliver PTX [146]. In a different work, a polymeric micelle based on thiolated Pluronic^®^ (Plu-SH) was fabricated, in which the micelle core was derived by functionalizing a disulfide bond on Pluronic^®^ F127 [147]. In a recent work, tumor suppressive gene p53 was linked to a PEGylated glycolipid-like polymer (P-CSSO) via disulfide bond to form P-CSSO/p53 complexes. Both in vivo and in vitro studies revealed that the PEGlylation resulted in a weakened macrophage uptake of the formulated system, and enhanced tumor accumulation [148]. A thiolated nanoparticle made of gelatin was developed by introducing a 2-iminothiolane group into the amino acid of gelatin which helped in faster transfection of the system by a NIH-3T3 cell line and a rapid nanoparticle disruption to release DNA [149]. While the ability of redox-responsive systems has been well established through a plethora of reports, the majority of such articles are focused on xenograft animal models or cell line studies that are unlikely to match in vivo human conditions. When compared to free drugs, most of these structures demonstrated early drug release in quantities that might be sufficient to induce harmful effects. A thorough analysis is also essential to evaluate the exact loaded drug dosage which is appropriate to achieve tumor regression without any side-effects with a rest time. Finally, dynamic structures often have issues in industrial scale up.

***(ii) Diselenide linkage-based systems.*** Selenium-based compounds are known to be sensitive to oxidative or reductive environments. Diselenide groups, when linked with polymeric scaffolds, undergo degradation upon oxidation to release their enclosed drugs. A few redox-responsive, polymeric NCs that function on this principle were recently investigated. A redox-responsive diselenide linkage containing a copolymeric system of Bi(mPEG-SeSe)-PCL,Bi(mPEG-SeSe)-PCL was fabricated to determine the DOX release from it. Both in vitro and in vivo studies revealed that the formulated delivery system was biocompatible and could further be used to develop more potent anticancer agents [150]. In another study, a DOX-loaded redox-responsive amphiphilic polymer, Bi(mPEG-PLGA)-Se2, with diselenide linkage, was formulated. In vitro studies using HeLa cells demonstrated a significantly higher drug release, which could be further used to synthesize other such anticancer drugs [151].

***Enzyme-responsive*.** The transformed enzyme expression profile serves as a vital marker to detect pathologically-challenged conditions, and is utilized extensively to develop enzyme-responsive DDSs. 

***(i) Esterase-responsive, polymeric systems.*** One polymeric delivery system that relies on the ester bond cleavage to release the drug to the targeted sites is made from esterase. Many esterase-responsive, polymeric systems have been developed by researchers so far, some of which shall be discussed here. A PAMAM-based, polymeric dendrimer was developed and linked to PTX via succinate bond. This PTX/PAMAM G4 dendrimers were readily hydrolyzed by esterase to release the free drug, aiding in effective cell internalization and reducing toxicity. Upon conjugation with PEG, these systems revealed an enhanced action which could be explored to develop more anticancer drugs [152]. To target inflammation, a novel twin-drug system, i.e., Dexamethasone-Diclofenac, was fabricated by esterification reaction and then encapsulated into polylactide (PLA) nanoparticles. The in vivo drug release was studied by employing esterase. It was revealed that esterase hydrolysis enhanced the drug release rate, and the synergistic activity of the two anti-inflammatory drugs led to higher inhibition of the TNF-α level than the free drug [153].

***(ii) MMP-responsive, polymeric systems.*** Elevated concentrations of matrix metalloproteinases (MMPSs) have been associated with many cancerous cells. This special type of enzyme could selectively cleave peptide linkages in between nonterminal amino acid sequences [154]. An MMP2-sensitive siRNA delivering, self-assembling, copolymeric system of polyethylene glycol-peptide-polyethylenimine-12, along with dioleoyl-snglycero-3-phosphoethanolamine (PEG-pp-PEI-PE), was developed. The linker was based on an octapeptide, GPLGIAGQ, which is extremely responsive to MMP2. This led to high tumor targeting of the developed system [155]. In a different study, N-(3-aminopropyl) methacrylamide (APM) and acrylaminde (AAM) were used to synthesize copolymeric nanocapsules with MMP-responsive peptide cross-linkers to cargo BSF and VEGF [156]. In a recent study, novel MMP2 sensitive nanoparticles were designed from copolymeric TPGS3350-pp-PLA along with TPGS-folate to deliver anticancer drugs [157]. An MMP 8-responsive, polymeric hydrogel was developed from a diacrylate-containing polyethylene glycol-based moiety, along with a cysteine-terminated peptide cross-linker (CGPQG↓IWGQC). The hydrogels were encapsulated with BSA, minocycline hydrochloride, and antibacterial peptide KSL to target chronic periodontitis and peri-implantitis [158].

***(iii) Cathepsin B.*** The tetrapeptide linkage Gly-Phe-Leu-Gly is mostly employed in the design of enzyme-responsive, polymeric systems, as this group is easily cleaved by cathepsin B, an lysosomal enzyme, which is typically overexpressed in tumor tissues [159]. A cathepsin B-responsive PEGylated gemcitabine-containing system was developed to target tumor microenvironments [160]. In another study, a peptide macromonomer was designed from BIM and cathepsin B substrate, which was then incorporated into copolymeric deblock system of DEAEMA/BMA and DEAEMA. The system was successful in the intracellular delivery of peptides [161].

***Hypoxia-responsive*.** Nitroaromatics, as well as azo-derivatives, are usually used to design hypoxia-responsive, polymeric drug and gene delivery systems. 

***(i) Azo-derivatives.*** Azo groups are easily reduced to amine derivatives under hypoxic cell conditions. This has been exploited in the development of bioreductive linkers, forming the majority of the drug releasing nanoparticles. A PAPD nanocarrier was developed using PEG, azobenzene, and polyethyleneimine, as well as 1,2-dioleyl-sn-glycero-3-phosphoethanolamine (DOPE), to deliver siRNA. This hypoxia-responsive nanoformulation showed effective silencing of green fluorescent protein (GFP) by the removal of PEG from the system [162]. In a different study, an azo derivative (AzP1) of an irinotecan analogue, SN-38, an FDA approved drug, was evaluated on various cancer cell lines. The tumor suppression potential of AzP1 was validated using a xenograft mouse model. The drug was not only shown to be capable of tumor specific activation and targeting, but was also extremely easy to synthesize [163]. Another group synthesized hypoxia-sensitive carboxymethyl dextran and an azo-bond-containing, black hole quencher 3 (BHQ3), self-assembling polymer conjugate to release DOX in a sustained fashion under physiological conditions. It was observed that the DOX release increased remarkably under hypoxic conditions because of the breakage of the azo bond. Both in vitro and in vivo studies revealed preferential tumor accumulation of the drug [164]. 

***(ii) Nitro-aromatic derivatives.*** Nitroaromatic derivatives are able to undergo single electron reduction to form amines by a series of reactions with nitro reductase that is coupled with naturally reducing agents that are intrinsically present in the tissues. Under normal oxygen conditions, these reactions are easily reversible, but for hypoxic cells, the nitro group of nitroimidazole is reduced to a free radical. In one study, a hypoxia-responsive, polymeric nanocarrier was synthesized; methoxypoly(ethylene glycol)-b- poly(glutamic acid) grafted with an imidazole derivative, 6-(2-nitroimidazole)hexyl amine along with pendent carboxylic group of mPEG-*b*-PLG. DOX was encapsulated into the micellar core of the formulation, and was tested on MCF-7 cell lines. It was observed that the DOX release was increased in hypoxic cells [29]. In another study, 2-nitroimidazole was linked to alkylated polyethyleneimine to form nanomicelle-like aggregations under aqueous conditions. This system was used to deliver surviving targeting siRNA to achieve tumor targeting [165].

***Temperature-responsive*.** In developing thermo-responsive, polymeric systems, it is important to take the note of critical solution temperature of the polymers. CST is the temperature at which polymers undergo a phase separation, moving from an isotropic state to anisotropic one [166]. Temperature-responsive, polymeric systems may be divided into two categories: Systems with negative temperature sensitivity, where the polymers swell due to the formation of hydrogen bonds at temperatures lower than lower critical state temperature (LCST) and collapse at temperatures above LCST [167]; and positive temperature-sensitive polymers which swell at temperatures above the upper critical state temperature (UCST) and collapse at temperatures below it [168]. Temperature-responsive polymers display a transition from a sol-gel form with varying temperature change; many hydrogels are capable of in situ gelation at body temperature [166]. Many chitosan thermo-responsive, polymeric systems have been developed. An in situ gelling, thermo-reversible, PEG-grafted chitosan hydrogel was fabricated to achieve the sustained release of drug [169]. Apart from chitosan, another animal-derived polymer, gelatin, has also been explored as a DDS. For example, an adjustable thermo-responsive hydrogel made from gelatin demonstrated a successful volume transition at physiological temperatures, and was reported as a promising vehicle for the delivery of many drugs [170]. Another study involved the development of an in situ gelling thermoresponsive hydrogel of chitosan/gelatin/beta-glycerol phosphate (C/G/GP) disodium salt which was utilized in nucleus pulposus regeneration. NP cells were cultured in the formulated hydrogels, and it was seen that the gene expression was modified in this system [171]. A copolymerized system of N,N-dimethylaminoethyl methacrylate (DMAEMA) and NIPAAM was synthesized to deliver genes. The complex was seen to be stable at body temperature, and demonstrated an enhanced transfection in OVCAR-3 cell lines [172]. In another study, a transdermal thermo-responsive, polymeric gel was developed from poly(N-vinylcaprolactam), PNVCL, which could transition to gel at 35 °C. Transdermal gel is extremely patient friendly, as it may be applied by the patients themselves. Acetamidophenol and etoricoxib were loaded into these gels to test their efficacy [173]. Pluronic, a famous PEO-PPO-PEO thermosensitive, polymeric system, is also capable of undergoing sol-gel transitions at body temperature [174]. Poly(N-isopoprylacrilamide) or pNIPAAm is a synthetic thermoresponsive polymer which is capable of forming gel at or near human body temperature [175]. The in situ gelling thermo-responsive hydrogel system was made from pNIPAAm and PAA that was used for drug delivery. Thermoresponsive ketoprofen-loaded nanofibers were synthesized using poly(N-isopropylacrylamide) (PNIPAAm) alone, ethyl cellulose (EC) alone, and a combination hybrid of both polymers [176].

***Glucose-responsive.*** Glucose-responsive, polymeric systems are promising candidates for regulating insulin delivery to the body in response to altered blood glucose levels, and thereby, in helping to maintain homeostasis. Many glucose-responsive, polymeric systems have been fabricated; they typically rely on the oxidation of glucose to gluconic acid via glucose oxidase (GOx). Nanocapsules incorporating chitosan, insulin, and the GOx enzyme were formulated as monodispersed microgels which could swell in a hypoglycemic environment by the protonation of chitosan, thereby releasing insulin for the treatment of diabetes [177]. Acryloyl crosslinked dextran dialdehyde (ACDD) nanoparticles with GOx functionalization were synthesized as novel glucose-responsive, as well as in vitro pH-responsive, delivery systems [41]. A GOx-containing hydrogel of poly(methacrylic acid-g-ethylene glycol) was developed that was capable of swelling at physiological pHs. When the system was exposed to glucose, there was an observed pH decrease, which led to the collapse of the hydrogel [178]. In a recent work, glucose-responsive polymersomes (Pep-PMS) were synthesized that could effectively bind to the ganglioside-monosialic acid receptors which are present in the epithelium of the intestine. These nanosystems were able to release the encapsulated insulin in hyperglycemic conditions based on GOx-induced H_2_O_2._ These orally-delivered, liver targeting systems were successful in regulating insulin delivery in rat models, and could be translated favorably into clinical praxis [179]. Lectin is a group of naturally glucose- and mannose-binding protein moieties. The most widely exploited molecule in this respect is Concanavalin A (Con A), a lectin family member which is capable of binding reversibly and specifically to glucose [180]. For example, Con A, grafted on glucosyloxyethyl methacrylate along with N,N′-methylene-bis-acrylamide (MBAAm), was used to fabricate glucose-responsive hydrogel [181]. In another study, Con A and dextran were polymerized by UV to create novel DDSs that could be utilized to deliver insulin in a closed-loop form [182]. Amylopectin and Con A were utilized to create a bio-responsive, self-assembling nanoparticle system to deliver insulin [183]. 

#### 2.3.2. External Stimulus

***Magnetic field-responsive*.** Magnetic field stimuli-responsive nanocarriers are composed of paramagnetic or super-paramagnetic substances enclosed in a polymeric scaffold. These systems are used extensively to develop therapeutics, diagnostics, as well as biomimetic actuators. Poly(ethylene imine) or Pluronic^®^ shells were cross-linked with magnetite to locally deliver siRNA [184]. A magnetic nanoparticle-embedded, chitosan microbead system was used to load vancomycin. The drug release was stimulated by alternating magnetic fields [185]. In a different study, ZnFe_2_O_4_ nanoparticles were coated with chitosan to cargo lidocaine, a local anesthetic [186]. A novel copolymeric system of poly[(2-succinyloxyethylmethacrylate)-b-(N-isopropylacrylamide)-b dimethyl aminoethylmetha- crylate) was fabricated by RAFT polymerization. Using succinyloxyethylmethacrylate, a triblock copolymeric system was coated on the surface of Fe_3_O_4_ nanoparticles to aid in the delivery of anticancer drugs [187]. A dual pH- and magnetic field-responsive magnetite coated with Eudragit^®^ S100 was fabricated and loaded with amoxicillin. In vitro studies demonstrated its usefulness as an antibacterial agent [188]. 

***Light-responsive*.** Light-responsive drug and gene delivery systems are designed by incorporating a linker that can be cleaved by light irradiation, or by using light-responsive molecular scaffolds like azobenzenes and spiropyrans [189]. 

***(i) Photo-responsive, polymeric systems derived from cleavable linkers.*** The introduction of a photocleavable linker into the polymeric backbone has helped in the design of photo-responsive, polymeric drug/gene delivery systems. One such linker is the nitrobenzyl ester linker. For example, a copolymeric polymerosome system was made from PAA attached to poly(methylcaprolactone) by a nitrobenzyl linker. The linker was extremely light-sensitive, and released fluorescein upon irradiation by an external light source [190]. Another study involved the attachment of bis-(3-aminopropyl)methylamine (AMPA) to pentaethylenehexamine (PEHA) via a nitrobenzyl linker that helped in gene- delivery after being irradiated at 350 nm [191]. In a recent study, PMMA-based photo-responsive microspheres and nanospheres were prepared using acrylate cross linkers that were made with two o-nitrobenzylester moieties. The formulation showed photo response at 366 nm, suggesting its wide range of application in constructing drug delivery and imaging formulations [192]. Another widely used linker to design photo-responsive systems is coumarin. A novel coumarin-functionalized copolymeric block of poly(ethylene oxide)-b-poly(n-butylmethacrylate-co-4-methyl-[7- (methacryloyl)oxyethyloxy] coumarin) (PEO-b-P(BMA-co-CMA) was used to deliver the anticancer drug 5-florouracil. Drug release was achieved at 254 nm [193]. 

***(ii) Spiropyrans based photo-responsive, polymeric systems.*** Spiropyrans, when irradiated with ultraviolet radiation, reversibly transforms from a nonionic state (hydrophobic) to an ionic polar hydrophilic isomer (merocyanine). A novel transdermal delivery system was synthesized by grafting poly(hydroxylethylmethacrylate) onto porous polymeric membranes and then modifying it with spiropyran [194]. In another study, polyglycerol micelles were modified with spiropyrans to aid in drug delivery upon exposure to UV irradiation [195]. In a recent study, a PDMAEMA and poly(methylmethacrylate) copolymeric system was functionalized with spiropyran to develop a photo-, thermo-, pH-, as well as CO_2_-responsive, polymeric system [196].

***(iii) Azo based photo-responsive, polymeric delivery systems***. The azo group can transform reversibly into a stable as well a polar cis state from a trans state upon UV irradiation. An azobenzene incorporating PEG-b-PAA copolymeric vesicle like system was designed. Upon irradiation with light, the azobenzene group aided in controlled dug release [197]. Another photo-responsive hydrogel system was developed by attaching azobenzene to cyclodextrin-decorated dextran. Upon irradiation, the system released the enclosed drug due to the transition of azo group from the trans to the cis phase [198]. 

***Ultrasound-responsive***. Ultrasound-responsive, polymeric systems have been used extensively to help in site-specific controlled drug delivery. Many novel nanodroplets, nanobubbles, nanomicelles, and nanogels have been developed as ultrasound-responsive, polymeric systems.

***(i) Nanogel***. To enhance the thromobolyis of clots, a urokinase type plasminogen activator was loaded in a copolymeric nanocapsule of chitosan and poly(ethyleneglycol). The drug release from the system was enhanced using a 2 MHz ultrasound [199]. In another study, a novel adriamycin gelatin nanogel delivery system was constructed by modification with a fluoride anion. At a frequency of 20kHz, the ultrasound triggered drug release [200].

***(ii) Micelles***. Pluronic micelles loaded with DOX were used as ultrasonically-responsive, polymeric DDSs. At a frequency of 20 kHz, maximum drug release was obtained [201]. In another study, a curcumin-encapsulating polymeric micelle was constructed from pluronic P123/F127. The site-specific release of curcumin was modified by ultrasound sonication, as confirmed by in vitro studies [202]. In another study, a PTX-incorporating, ultrasound-responsive, micellar system of PEG–PLLA, poly(ethylene oxide)-*co*-poly(L-lactide), or PEC-microbubble PCL (poly(ethylene oxide-*co*-polycaprolactone)) (PEO-*co*-PCL) was able to deliver PTX to ovarian as well as breast cancer cell lines upon exposure to a 1 MHz ultrasound [203].

***(iii) Nanobubbles.*** An ultrasound-responsive chitosan DOX nanobubble system was synthesized to deliver DOX following ultrasound exposure. It was observed that almost twice the amount of DOX was released, compared to the nonultrasound-responsive system [204]. Ultrasound-responsive, polymeric nanobubbles were constructed to deliver both siRNA and PTX to treat hepatocellular carcinoma [205]. In another, similar study, a new codelivery system, i.e., DOX- and shRNA-loaded PLGA and PEI nanobubbles, were designed to address DOX resistance in breast cancer [206].

***(iv) Nanodroplets.*** A stable nanodroplet encapsulating simvastatin was developed that could release the drug upon exposure to high intensity, focused ultrasound [207]. Nanodroplets are widely used to load 10-hydroxycamptotheci, a lipophilic anticancer drug [208]. In 2018, an ultrasound-responsive nanodroplet system was developed with four parts: Fe_3_O_4_ (for imaging), Folic acid, HCPT (for cancer therapy), and PFC as the core. Upon sonification, PFC vaporized, causing HCPT to be released [209]. In another study, DOX- and perfloropentane-incorporating, phase-changeable, lipid-PLGA, hybrid nanodroplets were constructed that released the drug upon exposure to low intensity, focused ultrasound (LIFU) [210]. Recently, an ultrasound-responsive nanodroplet was made from PFP/C_9_F_17_-PAsp(DET)/CAD/PGA-g-mPEG by incorporating an ultrasound-responsive contrast agent, fluorinated polymer, and a DOX prodrug that was successful in anticancer therapy [211]. 

***Electrical energy-responsive*.** Nanoparticles that are made of conducting polymers have been extensively used to deliver drugs. In situ polymerization and thermally-induced phase separation techniques were used to design nanofibrous scaffolds of polylactide with polyaniline to aid in osteogenesis [212]. Ibuprofen was loaded in MSN, which was then incorporated into a chitosan hydrogel system that was developed on titanium plate. It was seen that the release of ibuprofen increased with pH and electrical stimuli [213]. A phenytoin sodium-containing, electro-responsive hydrogel was developed to treat epilepsy. Stimulation from an electric field led to an increase in the ionization degree because of the poly(sodium-4-vinylbenzene sulfonate) that was present in the hydrogel [214]. A plethora of other electro-responsive, polymeric drug and gene delivery system have been investigated as well. Although these systems are not used in clinical practice, they do possess a lot of potential. 

## 3. Stimuli-Responsive, Polymeric Nanocarriers for Bioimaging

Polymer nanocarriers have outstanding advantages in terms of biocompatibility, tailoring capabilities, stability, biodegradability, and low cost of preparation, compared to inorganic nanomaterials [215]. Stimulus-responsive polymers (smart polymers) are highly efficient polymers which adjust to their environment. Responsive polymers can be sensitive to humidity, chemical compounds, temperature, pH, light intensity and wavelength, and electrical and magnetic fields. These materials may respond in various ways, e.g., altering transparency or color, becoming water conductive, and changing shape. Minor changes in the environment are usually enough to induce changes in the polymer’s characteristics. [216] Bioimaging is a noninvasive method of observing biological behavior over a given period of time which does not hinder the various life cycles, such as movement, respiration, etc., and helps to record the specimen’s 3D structure with minimal inconvenience. It is useful in linking subcellular structure observations and all tissues in multicellular organisms [217]. Many imaging techniques, such as optical imaging [218], magnetic resonance imaging (MRI) [219], nuclear imaging [220], ultrasound (US) [221], photoacoustic imaging (PA), single-photon computed tomography (SPECT), positron emission tomography (PET), etc., have been widely used in clinical applications. This section reports on the most recent developments in this area by analyzing in detail examples of nanoparticles that can be imaged using one or more imaging techniques.

### 3.1. Optical Imaging

**Figure 1:**
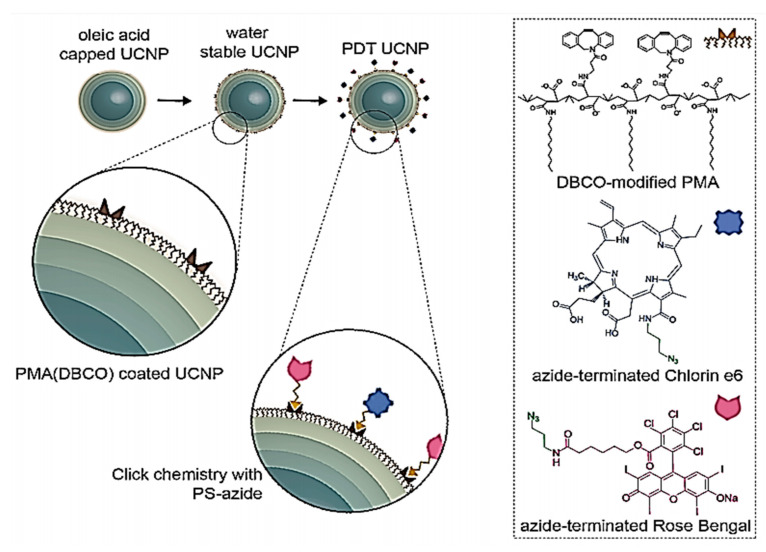
Depiction of the synthetic stages for the synthesis of UCNPs, their water exchange and two PSs functionalization (Rose Bengal and Chlorin e6). Reproduced from [232] with permission from Springer Nature, 2020.

Optical imaging is among the most commonly used methods in imaging [222]. Near-infrared (NIR) is a strategy that used by optical imaging systems because of the extremely low absorption of tissues in the wavelength of 700–1000 nm [223]. Small organic molecules with superior optical properties (high molar absorption, good photostability, and high fluorescent emissions in the NIR area) are fluorescent NIR probes such as cyanine compounds [224]. Such molecules, indeed, can be quickly degraded in aqueous media and in physiological environments are distinguished by a limited circulation time. In order to mollify these limitations, a wide variety of cyanines were incorporated into different polymer formulations with the goal of enhancing its bioavailability and durability. There are several studies of NIR fluorescent probes insertion in lipooligosaccharides [225], water-soluble carboxylated N-acylated poly(amino ester)-based comb polymers [226], supramolecular nanodiscs self-assembled [227], polymer micelle [228] and mitochondrion- and nucleus-acting polymeric nanoagents [229]. For instance, Yang et al. (2020) constructed a multi- and cascaded switchable polymer nanocarrier that self-assembled from nano polymers for imaging and anticancer treatment. Their nanocarrier is made up from PEG that transplanted an amphiphilic copolymer including hydrophobic poly (ortho ester) and a hydrophilic ethylenediamine-modified poly (glycidyl methacrylate) (PEG-g-p(GEDA-co-DMDEA)) [230]. In other research, J-aggregates of self-assembled amphiphilic cyanine dye FD-1080 and 1,2-dimyristoyl-Sn-glycerol-3-phosphocholine (1360 nm absorption and 1370 nm emission) were reported by Wang et al. (2019) [231]. Marinez et al. (2020) prepared a core@multishell nanoparticles (UCNPs) for phototherapy at 808 nm. First, they synthesized multicore of NaYF_4_:Yb_18%_Er_2%_@NaYF_4_:Yb_10%_@NaNdF_4_:Yb10%@NaYF4:Yb_10%_ and coated with amphiphilic DBCO-modified polymer PMA. After the polymer-coating of the UCNPs, they functionalized with two photosensitizers, Rose Bengal (RB) and Chlorin e6 (Ce6), Production of PDT nanoprobes with spatiotemporal resolution for 808 nm-gated intracellular reactive oxidative species (ROS) generation (Figure 1) [232].

### 3.2. Ultrasound Imaging

**Figure 2:**
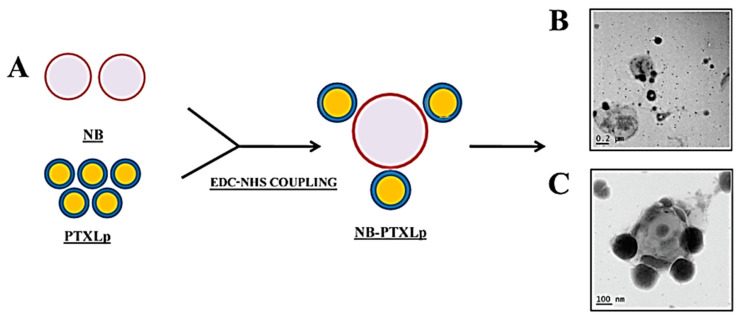
(**A**) Illustration depicting the formation of NB-PTXLp conjugates by EDC/NHS coupling, (**B**) Transmission electron micrograph of NB-PTXLp conjugate (scale bar—200 nm), (**C**) Transmission electron micrograph of NB/PTXLp conjugate (scale bar—100 nm). Reproduced from [234] with permission from American Chemical Society, 2019.

Ultrasound (US) Imaging is a low-cost, un-invasive, effective and real-time imaging method in which sound waves are transmitted to the patient’s body at 2 MHz or more [233]. The different tissues reflect these sound waves and are processed by a converter that converts these details into pictures. The most widely discussed cancer theranostic approach that incorporates cancer treatment With US imaging suggests the use of active oriented biodegradable polymers. Multifunctional PLGA Nanobubbles described by Hong et al. (2015) as theranostic agents. They integrated doxorubicin and P-gp siRNA into MCF-7 cancer cells and used this platform to conduct cellular ultrasound imaging [206]. In another research, Prabhakar et al. (2019) documented the production of nanobubble-paclitaxel liposome (NB-PTXLp) particles with size of 528 nm in cancer cells for ultrasound sensitive drug delivery and ultrasound imaging (Figure 2) [234]. Shang et al. (2019) also reported the preparation of ST68/PLA-PEG nanobubbles (NBs) for theragnosis and tumor imaging. These nanoparticles contain perfluoro propane gas, Span 60 and Tween 80 (ST68) surfactants and a block copolymer (PLA-PEG-NH_2_). These NBs showed a contrast strength of 3 dB with low loss of contrast signal after 10 min [235]. Also, some other methods for ultrasonic imaging tested such as high intensity-focused ultrasound (HIFU) [236], Passively targeted structures of natural polymers, such as alginate [236] or human serum albumin (HSA) [237] and Chemical development of gasses in the body in reply to tumor microenvironmental hallmarks.

### 3.3. Magnetic Resonance Imaging (MRI)

**Figure 3:**
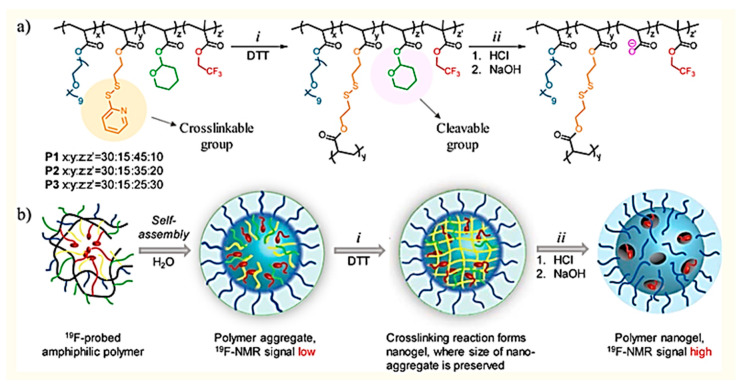
Design and synthesis of fluorinated probe. (**a**) Structure of polymer and nanogel. (i) Nanogel formation via crosslinking of PDS groups with DTT (ii) Cleavage of THP group in the presence of HCl and formation of negatively charged moiety with NaOH addition. (**b**) Schematic representation of preparation of fluorinated nanogel with decreased interior density. Reproduced from [242] with permission from American Chemical Society, 2019.

In MRI, a sufficient magnetic field is applied to arrange the magnetic moments of hydrogen atoms in tissues and disturbed by an external radiofrequency. After relaxation to their ground state, a radio frequency signal generated that is identified and converted into a picture [238]. The use of this strategy for cancer diagnosis is, however, limited due to low sensitivity or poor contrast [239]. The use of the MRI contrast agents (CAs) which are capable of altering the relaxation times of protons in different organs through their involvement with the external magnetic field is, therefore, necessary [240]. Low molecular weight complexes of these CAs cannot provide precise MRI imaging of the tumor. In addition, the need for large doses to have stronger tumor images dramatically raises the risk of systemic toxicity. Therefore the encapsulation or chelation of these CAs by polymeric nanoparticles (particularly smart polymers that react to tumor-specific stimuli such as acidic pH, overexpressed ROS, etc.) has shown great potential to address these disadvantages [238,241]. Experiments focused nowadays on the development of nanogels, polymersomes, micelles and so on in MRI imaging. Munkhbat et al. (2019) developed a system for covalently trapping nanoscopic states with an optimal degree of 19F substitutions. Major improvements in T2 relaxation times is achieved due to increased segmental mobility of side-chain substituents of stimulus-responsive polymer nanogel (Figure 3) [242]. In another paper, Bain et al. (2019) documented a combination of 2 diblock co-polymers made up from PEG and carboxylic acid terminated poly (2-methacryloxyethyl phosphorylcholine) (PMPC) (called polymersome). PMM28 magneto-polymersomes (PMM28Fe) showed a 6 ° C increase in temperature during magnetic hyperthermia in vitro, resulting in an intrinsic loss power (ILP) of 3.7 nHm^2^ kg^−1^ that offers the added potential for further tuning and functionalization for imaging and drug delivery purposes [243]. Aouidat et al. (2019) reported a new Gd(III)–biopolymer—Au(III) complex synthesis that acts as a key component of Gold core-shell nanoparticles (Gd(@AuNPs). They proved that Gd@AuNPs had some benefits to showing hepatocytes in the liver. In particular, these nanoconjugates provided a strong cellular absorption of several quantities of Gd@NPs into cells, while maintaining a T1 contrast within cells that provides robust in vivo detection using T1-weighted MR images [244].

### 3.4. Photoacoustic Imaging (PAI)

PAI or photoacoustic imaging is a most recently discovered and developed technique for visualization and applicable in cancer therapy. Photoacoustic effect is the basis of this method that generating localized heat and thermoelastic stress waves when tissues absorb a few nanoseconds of the optical pulse. It is a noninvasive method that provide better tissue penetration, higher contrast to ultrasound and improved spatial resolution than optical imaging [245]. Authors have so far mostly represented inorganic nanomaterials for PTT /PAI methods. Indeed, their photostability, lack of degradability and bio-toxicity, are increasingly troubling. Polymer-based systems tend to be the best option for solving biocompatibility and biodegradability challenges, and hence the recent publications focused on them [246,247]. Zhang et al. (2017) described a semiconducting donor-acceptor electron conjugated polymer nanoparticles (PPor-PEG NPs) with a light harvesting device that was designed for highly efficient photoacoustic imaging and phototherapy [247]. In another research, Lyu et al. (2016) designed an intraparticle molecular orbital engineering strategy to simultaneously boost the efficacy of polymeric nanoparticles in phototherapy and photoacoustic brightness for cancer therapy and in vivo imaging. They demonstrated the use of the strengthened SPN as the theranostic nanoagents allow for better photoacoustic imaging [248].

### 3.5. X-ray Computed Tomography 

X-ray computed tomography (CT) is amongst the most widely used approaches of noninvasive clinical imaging in modern medicine due to the high X-ray penetration potential. This technique utilizes ionizing X-rays to produce images by spinning an X-ray tube and a detector over a patient’s sides. The most frequently employed contrast agents for CT are iodinated small molecules, essentially 1,3,5-triiodobenzene derivatives [249]. Such molecules, however, have limited blood circulation, suffering from rapid clearance by the mononuclear phagocyte system. In this context, polymeric nanoparticles are an excellent means for preventing such inconveniences, increasing the pharmacokinetic effects of iodine molecules and reducing their renal removal [250]. In particular, research has focused on the production of AuNP polymeric nano-vehicles to enhance their tumor aggregation, their contrast of CT imaging, and also function as a radiosensitizer [251]. Jang et al. (2019) explored different self-assembled configurations of gold nanoparticle (AuNP)-block copolymer complexes generated in aqueous solution from a combination of Pluronic F127 and PE6200. AuNPs embedded in polymeric uni-lamellar vesicles may be used as imageable drug carriers, catalyst carriers, distributors of drugs or enzymes, and as nanoreactors [252]. Also, Shapoval et al. (2019) prepared nanoparticles of GdF3-structured, biocompatible, poly (4-styrene sulfonic acid-co-maleic acid): Eu^3+^(Tb^3+^). Their nanoparticles were very tiny (3 nm), with a narrow size range, and were observable by X-ray contrast imaging, making them potential useful for the simultaneous and comprehensive identification of diseased tissues [253].

### 3.6. Radionuclide Imaging

Radionuclide imaging (RT) generally involves computed tomography imaging (PET) for positron emission and computed tomography imaging for single-photon emission (SPECT). High energy from the positron annihilation of γ rays increases penetration efficiency compared to CT [254]; 18F is one of the best RT substances products, but it has a low half-life. To overcome this limitation, various research has focused on the development of specific polymeric nanovehicles [255]. For simultaneous PET imaging and combination therapy, Sun et al. (2018) reported a multifunctional polymeric carrier. They produced a farnesylthiosalicylate-based, triblock copolymer POEG-b-PVBA-b-PFTS (POVF), i.e., a nano polymer made from a poly(FTS) hydrophobic block, a hydrophilic block of poly (oligo(ethylene glycol) (POEG), and a middle block of poly(4- vinylbenzyl azide) (PVBA). PET imaging in the 4T1.2 tumor-bearing mice indicated rapid absorption and slow clearance of radiolabeled PTX/POVF nanomicelles in tumor tissues [256]. Interestingly, owing to its sensitivity at nanomolar and even picomolar rates, SPECT performed over 80% of all radio diagnostic scans. Thanks to their ideal nuclear decay properties, availability, and fair price, the most widely used gamma-emitting radionuclides are ^123^I, ^111^In, and ^99m^Tc. Sun et al. (2019) developed PEI-labeled gold nanoparticles (Au PENPs) with iodine-131 (^131^I) for radionuclide therapy and CT/SPECT imaging (PEI coated with PEG and then conjugated to it). Their method has proven to be effective for radionuclide therapy and CT/SPECT imaging of tumor cells in vivo and in vitro [257]. In another study, Goas et al. (2019) formulated gold nanoparticles grafted with hybrid poly(methacrylic acid) to improve the performance of systemic ^131^I-mediated RT on tumor-bearing mice [258]. This work was the first study of a simple and effective method focused on nanomedicine to reduce the dose of radioiodine required to achieve curability and RT imaging.

### 3.7. Multimodal Imaging 

Multimodal imaging describes the application of two or more imaging techniques in a single platform. Yang et al. (2019) developed indocyanine green (ICG)-conjugated and radionuclide iodine-125-labeled polymeric micelles (PEG-PTyr(125I)-ICG) by the self-assembly of an amphiphilic diblock polymer (ethylene glycol)–poly(l-tyrosine-125I)–(indocyanine black). This device showed a successful multifunctional nanoplatform with simple constituents for multimodality imaging of FL/ SPECT / PA [259]. Song et al. (2019) presented a multimodality imaging technique composed of MRI, magnetic particle imaging (MPI), and PA by MMPF NPs (a long-chain, semiconducting polymer (PCPDTBT) composed of (poly[2,6-(4,4-bis(2-ethylhexyl)-4H-cyclopenta[2,1-b;3,4-b′]-dithiophene)-alt-4,7(2,1,3−benzothiadiazole)]) and Fe_3_O_4_ nanoparticles) for imaging in vivo [260]. Hu et al. (2019) developed a platform of a gadolinium-conjugated responsive polymer (PFTQ-PEG-Gd NPs) for MR/PA/NIR-II tri-mod imaging and in vivo tumor phototherapy [261]. Au nanoparticles coated with PEG and conjugated with fluorescence polymers (PFBT and PFTBT) for bioimaging and in vivo X-ray-computed tomography were reported by Zhang et al. (2019) [262].

## 4. Stimuli-Responsive, Polymeric Nanocarriers for Theranosis (Physicochemical Properties)

A wide range of nanostructured materials such as carbon nanotubes, iron oxide nanoparticles, gold nanoparticles, and quantum dots can be applied for theranostic applications. However, theranostic materials such as iron oxide nanoparticles and carbon nanotubes in their pristine forms may induce oxidative stress and membrane destabilization, leading to cell death by apoptosis [263]; the integration of polymeric materials to theranostic probes is a potential remedy to overcome the these problems. Polymer-based theranostic modalities have garnered increasing research interest in the field of theranostic and nanomedicine. Polymers offer numerous advantages for theranostics, including nontoxicity, water solubility, biocompatibility, and the possession of multiple functional entities for the effective attachment of the theranostic agents to achieve target-oriented delivery of payloads [264,265,266]. The incorporation of polymeric nanostructures with targeting ligands like peptide, aptamer, antibody fragment, folic acid, saccharide, and polysaccharide for theranostic purposes has received broad attention recently [267]. These active ligands lead to the enhanced uptake, buildup, and internalization of the nanocarriers in cancer cells. Generally, polymer-based, multifunctional theranostic constructs comprise three main components: (i) the polymer backbone as a carrier; (ii) an imaging agent constituent such as iron oxide nanoparticles, quantum dots, and dyes for tumors imaging; and (iii) a targeting component such as aptamers, transferrin, and antibodies for targeted delivery [238]. This section provides a comprehensive overview of various classes of polymeric nanostructures such as liposomes, micelles, nanogels, and dendrimers that are widely employed to constitute theranostic probes.

### 4.1. Polymer Micelles

**Figure 4:**
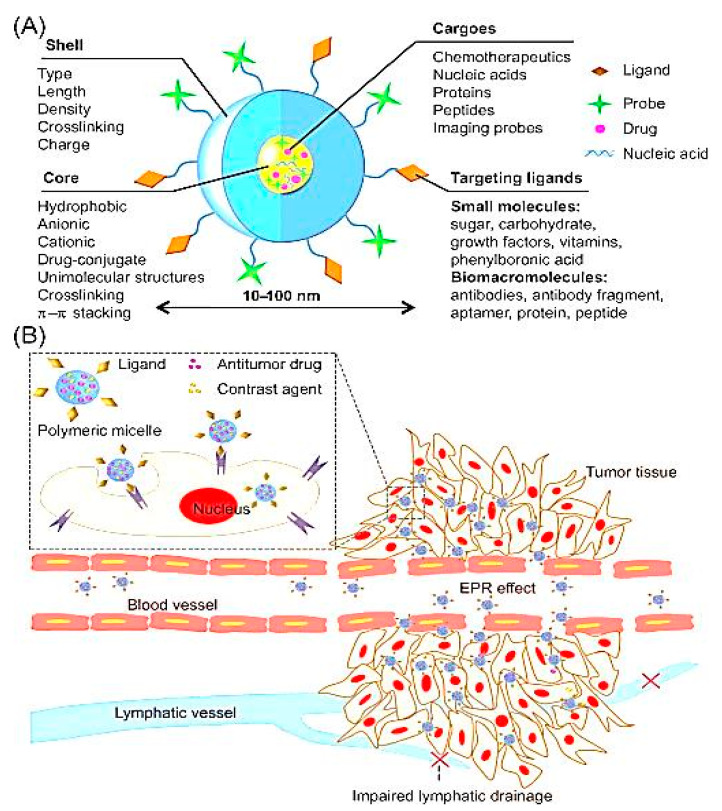
Polymeric micelles for cancer theranostics. (**A**) The composition of polymeric micelles. (**B**) Polymeric micelles target tumor tissues for cancer theranostics, and can be specifically accumulated in tumor tissues through the EPR effect for cancer diagnosis and therapy. EPR: Enhanced permeability and retention. Reproduced from [273] with permission from Elsevier, 2019.

Polymeric micelles (PMs) are self-assembled structures of amphiphilic polymers that contain both connected hydrophobic and hydrophilic moieties. The self-aggregation of amphiphilic polymers beyond a critical concentration of micelles assembles into a structure with a hydrophobic interior compartment and hydrophilic side chains extended outside [268,269]. A hydrophilic nanocarrier cannot effectively encapsulate most anticancer drugs due to their hydrophobic nature. Nevertheless, hydrophobic anticancer drug formulations can be efficiently loaded in micelles owing to the presence of a hydrophobic interior core. On the other hand, the hydrophilic shell of micelles substantially extends the stability of drugs in the blood by reducing phagocytosis and clearance through the kidney (Nguyen et al. 2016). PMs with an average diameter between 5–100 nm, and comprising a hydrophobic core and poly (ethylene glycol) shell, exhibit improved permeability and retention properties for the augmented cellular uptake of anticancer drugs in malignant tissues (Figure 4) [270,271,272]. 

Furthermore, the stimuli-responsive coordination of micelles facilitates the controlled release of drugs from micelle-based nanoplatforms. Therefore, intelligent PMs with stimuli-responsive behavior or tumor-targeted ligands have gained considerable research attention as novel carriers for drug delivery [274,275,276,277]. As a result, a wide variety of micelle-based, stimuli-responsive, theranostic modalities has been reported to target and eradicate cancer-causing cells [278].

Recently, Pourjavadi et al. (2020) used functionalized chitosan with poly(L-lactide) as an amphiphilic entity to prepare micelle in aqueous solution. The temperature-responsive, polymeric chain consisting of poly(acrylamide) and poly(N-isopropyl acrylamide) was subsequently embedded onto a chitosan- poly(L-lactide) composite to bestow these functionalities upon the micelles. The integration of gold nanorods onto micelles via gold-thiolate complexation induces photo-responsiveness feature to the nanocarrier. The in vitro drug release profile showed a 38% release of hydrophilic anticancer drug paclitaxel following exposure to NIR light at a determined time [279]. Zhu et al. (2019) developed novel, pH-responsive, retinal/indocyanine green (ICG) micelles as an all-in-one theranostic candidate for the treatment of cancer [280]. 

The synthesized homogenous micelle conjugates with spherical shape showed effective anticancer potentiality both in vivo and in vitro, with adequate biosafety. The loading efficiency of retinal/indocyanine green was recorded to be 9.23%, indicating its outstanding loading capacity. Amphiphilic hyaluronan-SS-poly(ε-caprolactone) diblock copolymers (HA-SS-PCL) were developed and employed as multifunctional nanocarriers for the diagnosis and management of tumor nanocarriers [281]. On one hand, HA shells containing theranostic nanoparticles possessed a higher affinity to CD44 expressed on the surface of malignant cells, leading to the accumulation of elevated levels of drugs. On the other hand, disulfide bonds connected HA-SS-PCL nanocarriers exhibited a reduction of the agent-triggered release of doxorubicin under a given level of glutathione. Furthermore, the encapsulation of superparamagnetic iron oxide and doxorubicin into the core of the micelles facilitated the diagnosis and treatment of targeted cancer cells. Notably, 100% release of DOX was achieved from HA-SS-PCL micelles within a period of 12 h under the reductive environment of glutathione (10 mM), while DOX release was shown to be about 40% within 24 h in the nonreductive conditions. Characterization analysis confirmed that the DOX-encapsulated HA-SS-PCL micelles were internalized in HepG2 cells through a receptor-assisted mechanism between CD44 receptor and hyaluronan. Cell apoptosis and MTT assay revealed prominent anticancer activity of the DOX-bearing HA-SS-PCL micelles against HepG2 cells compared to the reduction-insensitive HA-PCL micelles under identical conditions. Thus, the newly developed HA-SS-PCL block copolymers presented promise as versatile, tumor-targeting theranostic nanocarriers [281]. Shao et al. (2019) utilized polymer-based micelles to control indocyanine green (ICG) J-aggregation in a highly effective and rapid way. Besides a simple entrapment, the fabricated polymer micelles functioned as a promising host prototype to induce ICG J-aggregation by a combination of hydrophobic electrostatic interactions. The ICG J-aggregate remained intact in the polymer supramolecular assembly intracellularly due to efficient host–guest interactions. These features make this hierarchical assembly between ICG J-aggregate and the micelle polymer promising biomedicines for cancer phototheranostics. Moreover, these polymer micelles were modified by introducing doxorubicin for better therapeutic effect and covalent coupling of DNA aptamer for tumor targeting. In this way, this multifunctional, micellar-based nanomedicine revealed superior therapeutic potential for tumors, which were shown to be fully eradicated with no toxicity effects or reemergence up to 24 days following the treatment [228].

### 4.2. Polymeric Liposomes

Liposomes are spherical vesicles that consist of phospholipids forming a bilayer structure upon dispersion in water. Liposomes are one of the oldest, nonimmunogenic, and most successful biologically-inert, nanosized platforms for the delivery of drug molecules [282]. Liposome-based theranostics offer many benefits such as easy preparation, biocompatibility, and the ability to encapsulate hydrophobic as well as hydrophilic agents [283]. 

Hydrophobic drugs are incorporated into the liposomal bilayer membranes, whereas hydrophilic drugs can be encapsulated into the aqueous interior core. Nevertheless, the rapid removal of these liposomes by the phagocytic system is a major problem, decreasing their ability to reach target tissues. Therefore, surface modifications of liposomes are necessary to capitalize upon their efficient functioning. Modifications to the liposome surface using molecules such as polyethylene glycol and glycolipid considerably increase the circulation span of liposomes [284]. They also circumvent liposomal opsonization, and thus rescue from the mononuclear phagocytic system. Consequently, the longer circulation lifespan profoundly extends the theranostic potential of liposome-driven drug preparations in the body [285].

The FDA approved doxorubicin-loaded PEGylated liposome as the first nanomedicine in 1995 to treat AIDS-related Kaposi’s sarcoma. Since then, liposomal theranostic has been widely advocated for the diagnostic and therapeutic needs of numerous disorders including hepatitis, leukemia, breast cancer, macular degeneration, and fungal diseases [286,287,288]. In addition, many clinical trials are currently in progress [289]. Zhao et al. (2016) demonstrated that a pH-sensitive peptide (H7K(R2)2)-modified tumor-targeted liposome efficiently (over 80%) released the encapsulated DOX at a pH of 6.5. The pH-responsive DOX-containing liposomes also showed better tumor-controlling capacity than DOX-loaded liposomes without modification of the pH-responsive peptides [290]. In a recent study, Mansoori and coworkers (2020) tested the ability of hyaluronic acid-modified 5-fluorouracil (5-FU) -loaded, nanosized liposomes against colorectal cell lines (CD44-expressing) and a hepatoma cell line (non-CD44 expressing) [291]. An MTT assay revealed target-oriented tumor cell death in a time-dependent manner based on the expression of CD44. Cells treated with the newly-developed, liposomal-based, theranostic system exhibited significantly reduced oncogenic microRNA, mRNA, and colony formation, while tumor suppression was meaningfully increased in the treated group. Similarly, a new type of Arginine_8_-Glycine-Aspartic acid-modified, specific liposomal system was engineered for the separate encapsulation of emodin and daunorubicin [292]. The two-targeted liposomes were then combined to prevent tumor metastasis and disrupt vasculogenic mimicry channels. The results indicated potent toxicity and effective inhibitory activity of the combined liposomal system against the MDA-MB-435S cell lines (a highly intrusive type of breast cancer cells) and the metastasis of tumor cells, as well as the formation of vasculogenic mimicry channels. Insight into the mechanism suggests the downregulation of some metastasis-associated proteins, including VE-cad, HIF-1α, TGF-β1, and MMP-2 by the action of Arginine_8_-Glycine-Aspartic acid-modified daunorubicin and emodin liposomes. The specifically modified liposomes also facilitated the selective accumulation of chemotherapeutic drugs at the tumor site to exhibit direct antitumor activities, consequently providing a promising theranostic system for breast cancer [292].

### 4.3. Polymeric Dendrimers

Dendrimers are three-dimensional, hyper-branched polymeric structures that are increasingly being used in a range of drug and gene delivery applications [293]. In contrast to traditional polymer nanovectors, these polymeric, nanosized macromolecules exhibit low poly-dispersity, well-defined chemical structure, and diverse surface functionalities. Dendrimers can be formed by two different techniques, i.e., the explicitly divergent and convergent synthetic methods [294]. In the divergent technique, the dendrimers are assembled from the core and propagate outward into different generations or branches. The branches of the dendrimer originate in the convergent method and congregate towards the center, producing the core of the dendrimer [295]. As an intriguing class of drug nanocarriers, dendrimers polymers offer many beneficial features such as stimuli-responsiveness, high drug loading capacity, and target-specific drug delivery. The loading of drugs is achieved by attachment to the terminal branches or by conjugating the drug into the central core of the dendrimer polymer [296]. The presence of abundant functional moieties at the terminal ends promotes its conjugation with targeting ligands and imaging modalities to constitute multipurpose therapeutic/theranostic dendrimers. 

**Figure 5:**
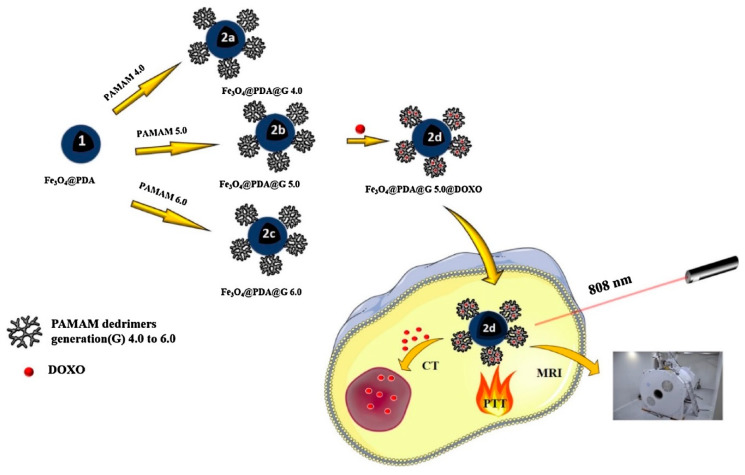
The schematic synthesis presentation of theranostic nanoplatform based on the Fe3O4@PDA@G 4.0–6.0 nanoparticles and its application in combined CT-PTT therapy on HepG2 cells. Reproduced from [300] with permission from Elsevier, 2019.

Owing to their unique properties such as nanoscopic size, monodispersity, biocompatibility, tunable size, reproducibility, and multiple functional groups at the periphery, dendrimers hold excellent potential as drug nanocarriers for theranostic applications. The cytocompatibility of dendrimers could be further augmented by amino acid, glycosylation, acetylation, and PEGylation functionalization to overcome their cationic toxicity to normal cell lines [297]. A DNA-assisted theranostic dendrimer displayed several fascinating characteristics, such as cytocompatibility, robust stability, satisfactory drug loading, and improved cellular internalization efficacy. Moreover, the engineered dendrimer structure also revealed an antitumor effect against acute lymphoblastic leukemia cells [298]. pH- and redox potential-responsive PEGylated dendrimers exhibited high cellular internalization and anticancer activities towards A549 cell lines. The antitumor efficiency of the as-designed theranostic dendrimers demonstrated platinum distribution, NIR tumor imaging, and good pharmacokinetics in A549 xenograft tumor-harboring mice [299]. Jędrzak et al. (2019) integrated polydopamine (PDA)-coated magnetite nanoparticles and PAMAM dendrimers to synthesize novel and multifunctional nanoplatforms. The designed PAMAM dendrimer G 5.0 functionalized nanocarriers exhibited a high drug-encapsulating capacity for doxorubicin hydrochloride, and were successful applieded in the photo- and chemothermal treatment of the human hepatoma HepG2 cell line (liver cancer cells), even at lower nanoparticle concentrations (Figure 5) [300].

The coordinated use of dendrimers with magnetic nanoparticles resulted in a versatile hybrid nanosystem-based drug delivery nanosystem for cancer treatment. Substituted silicon naphthalocyanine (SiNc) was transformed into a biocompatible nanoplatform (SiNc-NP) by encapsulating SiNc into the hydrophobic core of the polyethylene glycol-modified polypropylene imine dendrimer G5 surface. Experimental results showed that SiNc-mediated phototherapy effectively destroyed chemotherapeutic-resistant ovarian cancer cell lines. Additionally, the solid tumors were completely eradicated by treatment with SiNc-NP in combination with NIR radiation exposure without cancer reappearance [301]. Fan et al. (2019) developed a powerful theranostic, nanostructured platform based on copper(II)-complexed poly(amidoamine) dendrimers G5 for the treatment of tumor metastasis. It was found that the copper(II) complexes were capable of inhibiting the propagation of various tumor cells, accompanied by the induction of substantial cancer cell apoptosis, and thus, hold strong potential for suppressing the proliferation of different types of cancer cells. An engineered polyvalent nanosystem comprising iron oxide nanoparticles modified with folic acid-polyamidoamine dendrimers also exhibited a high accumulation of 3,4-difluorobenzylidene-curcumin (a potent anticancer agent), along with a promising anticancer effect [270].

### 4.4. Polymeric Nanocomposite Nanogels

Nanogels are physically or chemically cross-linked polymeric networks exhibiting attributes such as biocompatibility, mechanical strength, high water absorption capacity, good dispersion stability, structural permeability, and fast response to external stimuli [302]. These properties along, with high drug loading capability, make nanogels highly alluring nano-vehicles for drug and gene delivery applications. The high drug loading capacities of nanogels may be ascribed to the occurrence of high-water contents that constitute large cargo spaces inside the nanogel network [303]. This water content also contributes to their good biocompatibility compared with other polymer nanovectors including liposomes, dendrimers, and micelles. Drugs can be loaded onto nanogels by various approaches, such as self-assembly, covalent coupling, and physical entrapment. Likewise, the encapsulated drug can be unloaded via degradation, diffusion, and alterations in ionic strength, pH, and temperature [304]. Among the various theranostic nanoplatforms, polymeric nanogels are regarded as the most imperative owing to their exceptional features such as water content, good cytocompatibility, drug-encapsulating capacity, stimuli sensitivity, and the presence of multipurpose functional groups that ensure the conjugation of ligands. 

**Figure 6:**
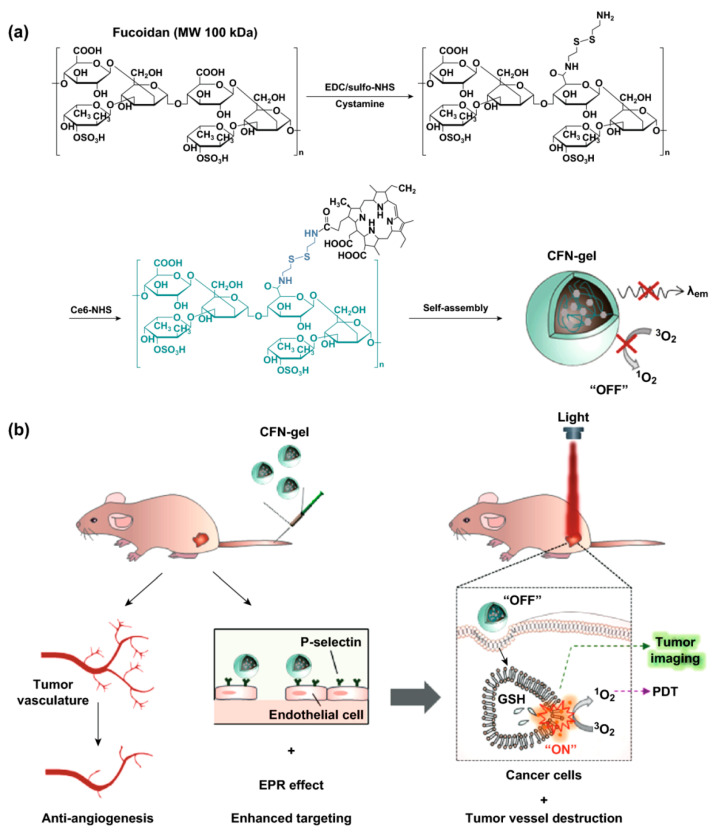
(**a**) Synthetic steps of Ce6–fucoidan theranostic nanogel (CFN-gel), (**b**) schematic illustration of CFN-gel and its mode of action. Reproduced from [305] with permission from Springer Nature, 2020.

A vast number of reports have supported the role of polymeric nanogels as promising theranostic carriers for the treatment of cancer with high specificity and efficiency. Recently, Cho et al. (2020) [305] developed a new, fucoidan, polysaccharide-based, nanogel theranostic platform (CFN-gel) containing a fucoidan backbone, photosensitizer, and redox-sensitive linking agent to achieve complete eradication of cancer cell proliferation (Figure 6).

**Figure 7:**
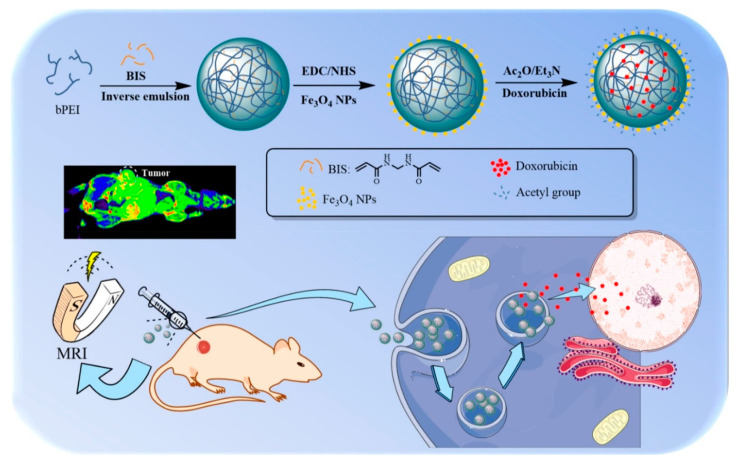
Schematic representation of the synthesis of Fe_3_O_4_/PEI-Ac NGs/DOX complexes for MR imaging-guided chemotherapy of tumors. Reproduced from [306] with permission from American Chemical Society, 2020.

The as-synthesized theranostic nanogel system exhibited a nanomolar affinity to P-selectin that was overexpressed on the surface of a variety of tumor cells, including neovascular endothelial cancer cells. Upon systemic administration, the CFN-gel displayed no phototoxicity because of the singlet oxygen generation and aggregation-triggered self-quenching in its fluorescence. Due to its nanomolar affinity towards endothelial growth factors, CFN-gel also ensured substantial anticancer activity without light irradiation, revealing fucoidan-based nanogels as novel and specific theranostic materials for cancer treatment. Polyethylenimine (PEI)-based versatile nanogels (NGs) were fabricated, for the first time, by adopting an inverse miniemulsion polymerization, subjected to modification with ultra-small iron oxide nanoparticles, and used to load doxorubicin, an anticancer drug (Figure 7). 

The fabricated nanoplatform possessed better colloidal stabilization, good water dispersibility, exceptional drug accommodating efficacy, and pH-responsive doxorubicin release with enhanced release in an acidic environment. With regard to drug-free nanogels exhibiting good biocompatibility, the doxorubicin-incorporated hybrid nanovehicles showed remarkable therapeutic potential with an efficient uptake rate by cancer cells. Given these desired features, the growth of a tumor was completely suppressed by the newly-designed hybrid polymeric nanogels system [306]. Gao et al. (2020) reported the development of a novel class of decomposable magnetic redox- and temperature-responsive polymer/iron oxide nanocomposite nanogels (NCGs) by the inverse emulsion method, and applied as nanovehicles for targeted drug delivery. Apart from high superparamagnetism, the resultant polymeric nanogel system also presented a noticeable redox- and rescindable thermo-responsiveness, and the release 5-fluorouracil antitumor drug was easily tuned by the redox environment, the external temperature of the media, or both. Additionally, the negligible cytotoxicity of the synthesized nanocomposite nanogels underlined its considerable potential to serve as a multifunctional nanoplatform for drug delivery [307].

## 5. Applications in Tissue Engineering and Regenerative Medicine

The fields of tissue engineering and regenerative medicines are parts of the broad field of life sciences, and usually make use of both biological and engineering aspects in the reconstruction of injured or diseased cells, tissues, and organs [308]. These field have shown significant relevance for biomedical applications [309]. Biopolymers and their combinations have played crucial roles and are amongst the most favorable potential constituents for the development of tissue engineering materials [310]. Also, stimuli-responsive biopolymer-mediated systems have found significant success in the development of biocompatible and biodegradable materials used for tissue/bone engineering and regenerative medicine applications. 

Moreover, the physical and mechanical characteristics of the materials can be modified by mixing and establishing porous assemblies and films with desired architectures [311,312]. Biopolymers including chitosan, collagen, alginate, and others have been extensively used for the production of stimuli-sensitive scaffolds and associated constructs [313,314,315,316]. Tissue engineering mediated materials are highly dependent upon cell-associated constructs that provide cellular linkage, progression, propagation, disparity, and relocation [317,318].

Apart from polymer conjugated systems, lipid-based systems have also shown significant potential in the production of regenerative medicines for cancer therapy [319] and other disorders or diseases [320]. In a study, researchers developed stimuli-responsive and chitosan-based by-functional polymerized nanocarriers to act as a potential material for drug delivery and tissue engineering applications. The polymeric nanoparticles (PNPs) were tested for various altered environmental pH and redox conditions, i.e., similar to those of the environmental conditions within the cancer cells. The PNPs were found to be biodegradable and biocompatible; however, the PNPs got deassembled in the presence of both stimuli. Nonetheless, with such characteristics, these PNPs could potentially be used as nanocarriers for the targeted delivery of chemotherapeutics [321].

Some researchers developed a triple stimuli-responsive carrier system, i.e., with pH, GSH, and enzyme sensitivity, for the efficient delivery of K-DOX-NPs. The K-DOX-NPs exhibited high drug loading efficiency and were found to be stable in an aqueous medium. It was observed that the overexpression of trypsin led to the creation of peptide bonds inside the K-DOX-NPs, and simultaneously enhanced the drug release at targeted sites in an A549 microenvironment. A cytotoxicity assay revealed that the K-DOX-NPs effectively inhibited the propagation of tumor cells. The K-DOX-NPs exhibited excellent biocompatibility and antitumor effects with minimal or no adverse effects, as well as sustaining blood circulation, making them a potential approach in cancer therapy [322]. Liu et al. evaluated the therapeutic efficacy of calcium alendronate-coated scaffolds. The coated scaffold composites exhibited superior cyto-compatibility, cell linkage aspects, upregulated osteogenic associated gene expression, protein levels, alkaline phosphatase (ALP) activity, and calcium deposition of an Adipose-derived mesenchymal stem cell (ADSC). It was concluded that the coated scaffolds exhibited osteogenic differentiation against ADSC due to improved integrin conjugation and FAK/ERK activation. Thus, the coated scaffolds could act efficiently in bone tissue engineering applications [323]. Biopolymer-mediated, stimuli-sensitive, switchable conditions were reported to play a shape-memory role as an output in the presence of two significant input signals, i.e., salt concentration and environmental temperature. These studies were meant to be functional in the presence of gelatin-conjugated hydrogel systems. The hydrogels displayed similar mechanical activities to those of soft tissues, with greater swelling abilities. The presence of gelatin significantly affected the hydrogel, and was responsible for the formation or deformation of a helical shape. Also, the study showed the potential activity of various chaotropic and kosmotropic salts which regulated the gelatin helicalization or conformational alterations [324].

Patel et al. modified the surface of biopolymer-mediated nanofibers with carbon nanotubes (CNTs), improved the topography of the CNTs, and evaluated their angiogenic, inflammatory, and bone rejuvenation activities. The CNTs-coated nanofibers, when administered subcutaneously to rat models, potentially downregulated cytokine levels and accumulations of macrophage, thereby decreasing inflammation. Also, the modified nanofibers supported angiogenesis and exhibited significant bone/tissue restorative activity against calvarium bone deficient models. Moreover, the in vivo results showed that the modified nanofibers exhibited greater bone mineralization and upregulation of osteogenic symptoms. Regenerative medicine-based approaches for the effective and targeted delivery of biomaterials by means of stimuli-responsive biopolymers have significantly increased in number in recent years [325]. In this context, Li et al. developed dual stimulus (redox and pH) -responsive, polymeric (keratin) nanoparticles for the targeted delivery of doxorubicin (DOX). Initially, the keratin-coated DOX nanoparticles (K-DOX-NPs) were fabricated using a desolvation method followed by chemical cross-linking with the drug entity. The K-DOX-NPs demonstrated both pH- and glutathione- (GSH) responsive aspects. The K-DOX-NPs accumulated in the tumor microenvironment due to the enhanced permeability and retention (EPR) effect, and executed a negative-to-positive conversion of surface charge. The K-DOX-NPs were found to be compatible, exhibiting potential activity against A549 human lung carcinogenic cells and promoting nitric oxide (NO) release, and thus, could serve as a potential chemotherapeutic tool for regenerative medicine [326].

In another study, Rebelo et al. developed and optimized a glycan-conjugated, collagen-mediated hydrogel that contacted with and controlled the disparity of neuronal cultures. To evaluate the activity of the conjugated materials, collagen polymers underwent amine-based reactions with maltose and lactose to eradicate the pyranosidic assembly of both. The glycan-conjugated, collagen-mediated, hydrogel inhibited astrocytic propagation (for up to two weeks) and brought about an upsurge of sialylation while decreasing fucosylation. Thus, the results indicated that modified hydrogels could serve as regenerative medicines for central nervous system-associated disorders [327].

## 6. Conclusions and Future Perspectives

Nanomedicine and theranostics are emerging research fields that incorporate the benefits of diagnosis and therapeutic agents into a single nanocarrier commodity. The fabrication of multifunctional nanostructured materials facilitates the target-oriented delivery of therapeutic or imaging agents for simultaneous diagnosis and theranostic applications. These newly developed nanocarriers not only contribute to effective treatments, but also ensure the instantaneous real-time monitoring of therapeutic responses. The rapidly developing field of stimuli-responsive, polymeric nanocarrier systems has already shown their efficiency in drug or gene delivery to the desired cells or tissues. Additionally, with better understanding of biological variances between diseased and healthy cells or tissues, and developments in nanomaterial approaches, there is potential to progress in the field of stimuli-responsive nanocarrier systems to achieve the improved targetability and biodistribution of drugs and genes. Also, stimuli-responsive, polymeric nanocarriers might show synergistic activitiy because of the simultaneous presence of an active polymer matrix and a stimulus component. Although various drug-/gene-incorporated, stimuli-responsive, polymeric nanocarriers have been established for different biomedical applications, there are some limitations that should be addressed in the future; these include the safety of the applied material or its modified compounds, the toxicity profile, alterations of moieties in various disease conditions, and variations in the in vitro and in vivo efficacy in the presence of different stimuli. This review also outlined several stimulus-responsive nanopolymers for developing imaging techniques. The classification of parts was based on the imaging method and nanopolymer kind. Objectively-constructed nanocarriers can be designed to deliver more effective therapy while reducing toxicity with a combination of stimulation sensitivities for bio imaging. There has been substantial progress in the development of nanocarrier systems for imaging, and further research will continue to broaden their abilities. The integration of polymers to nanoscale materials such as carbon nanotubes, iron oxide nanoparticles, gold nanoparticles, and quantum dots has significantly contributed to the field of theranostics and nanomedicine. Polymer-based nanomaterials, namely liposomes, micelles, nanogels, and dendrimers, offer numerous advantages for theranostics, including nontoxicity, water solubility, biocompatibility, and the possession of multiple functional entities for the effective attachment of theranostic agents to achieve the target-oriented delivery of payloads. However, it is equally important that these nanocarriers reach the specific site, perform their therapeutic function, and are then metabolized or excreted from the body. Therefore, several parameters, i.e., the charge, size, and surface functionalization of the polymeric nanostructures, should be optimized to ensure durable blood circulation and prevent renal clearance, as well as to evade capture by the reticuloendothelial system. In conclusion, significant efforts are needed for the successful and broad use of polymeric nanomaterials in drug delivery, imaging, and cancer theragnosis.

## References

[1] Kundu J.K., Surh Y.-J. (2010). Nrf2-Keap1 Signaling as a Potential Target for Chemoprevention of Inflammation-Associated Carcinogenesis. Pharm. Res..

[2] Mura S., Nicolas J., Couvreur P. (2013). Stimuli-responsive nanocarriers for drug delivery. Nat. Mater..

[3] Lee E.S., Oh K.T., Kim D., Youn Y.S., Bae Y.H. (2007). Tumor pH-responsive flower-like micelles of poly(L-lactic acid)-b-poly(ethylene glycol)-b-poly(L-histidine). J. Control. Release.

[4] Neri D., Supuran C.T. (2011). Interfering with pH regulation in tumours as a therapeutic strategy. Nat. Rev. Drug Discov..

[5] Chawla J.S., Amiji M.M. (2002). Biodegradable poly(ε-caprolactone) nanoparticles for tumor-targeted delivery of tamoxifen. Int. J. Pharm..

[6] Deng Z., Zhen Z., Hu X., Wu S., Xu Z., Chu P.K. (2011). Hollow chitosan–silica nanospheres as pH-sensitive targeted delivery carriers in breast cancer therapy. Biomaterials.

[7] Luo F., Fan Z., Yin W., Yang L., Li T., Zhong L., Li Y., Wang S., Yan J., Hou Z. (2019). pH-responsive stearic acid-O-carboxymethyl chitosan assemblies as carriers delivering small molecular drug for chemotherapy. Mater. Sci. Eng. C.

[8] Saha B., Choudhury N., Seal S., Ruidas B., De P. (2019). Aromatic Nitrogen Mustard-Based Autofluorescent Amphiphilic Brush Copolymer as pH-Responsive Drug Delivery Vehicle. Biomacromolecules.

[9] Cheng R., Meng F., Deng C., Klok H.-A., Zhong Z. (2013). Dual and multi-stimuli responsive polymeric nanoparticles for programmed site-specific drug delivery. Biomaterials.

[10] Pan Y.-J., Chen Y.-Y., Wang D.-R., Wei C., Guo J., Lu D.-R., Chu C.-C., Wang C.-C. (2012). Redox/pH dual stimuli-responsive biodegradable nanohydrogels with varying responses to dithiothreitol and glutathione for controlled drug release. Biomaterials.

[11] Torchilin V.P. (2014). Multifunctional, stimuli-sensitive nanoparticulate systems for drug delivery. Nat. Rev. Drug Discov..

[12] Wilson D.S., Dalmasso G., Wang L., Sitaraman S.V., Merlin D., Murthy N. (2010). Orally delivered thioketal nanoparticles loaded with TNF-α–siRNA target inflammation and inhibit gene expression in the intestines. Nat. Mater..

[13] Cheng R., Feng F., Meng F., Deng C., Feijen J., Zhong Z. (2011). Glutathione-responsive nano-vehicles as a promising platform for targeted intracellular drug and gene delivery. J. Control. Release.

[14] Tian Y., Guo R., Jiao Y., Sun Y., Shen S., Wang Y., Lu D., Jiang X., Yang W. (2016). Redox stimuli-responsive hollow mesoporous silica nanocarriers for targeted drug delivery in cancer therapy. Nanoscale Horiz..

[15] Mutlu-Agardan N.B., Sarisozen C., Torchilin V.P. (2020). Cytotoxicity of Novel Redox Sensitive PEG2000-S-S-PTX Micelles against Drug-Resistant Ovarian and Breast Cancer Cells. Pharm. Res..

[16] Nguyen M.M., Carlini A.S., Chien M.-P., Sonnenberg S., Luo C., Braden R.L., Osborn K.G., Li Y., Gianneschi N.C., Christman K.L. (2015). Enzyme-Responsive Nanoparticles for Targeted Accumulation and Prolonged Retention in Heart Tissue after Myocardial Infarction. Adv. Mater..

[17] de la Rica R., Aili D., Stevens M.M. (2012). Enzyme-responsive nanoparticles for drug release and diagnostics. Adv. Drug Deliv. Rev..

[18] Basel M.T., Shrestha T.B., Troyer D.L., Bossmann S.H. (2011). Protease-Sensitive, Polymer-Caged Liposomes: A Method for Making Highly Targeted Liposomes Using Triggered Release. ACS Nano.

[19] Radhakrishnan K., Tripathy J., Gnanadhas D.P., Chakravortty D., Raichur A.M. (2014). Dual enzyme responsive and targeted nanocapsules for intracellular delivery of anticancer agents. Rsc Adv..

[20] Zhu L., Wang T., Perche F., Taigind A., Torchilin V.P. (2013). Enhanced anticancer activity of nanopreparation containing an MMP2-sensitive PEG-drug conjugate and cell-penetrating moiety. Proc. Natl. Acad. Sci. USA.

[21] Kessenbrock K., Plaks V., Werb Z. (2010). Matrix metalloproteinases: Regulators of the tumor microenvironment. Cell.

[22] Zhu L., Kate P., Torchilin V.P. (2012). Matrix Metalloprotease 2-Responsive Multifunctional Liposomal Nanocarrier for Enhanced Tumor Targeting. ACS Nano.

[23] Chen W.-H., Luo G.-F., Lei Q., Jia H.-Z., Hong S., Wang Q.-R., Zhuo R.-X., Zhang X.-Z. (2015). MMP-2 responsive polymeric micelles for cancer-targeted intracellular drug delivery. Chem. Commun. (Camb. U.K.).

[24] Lee S.J., Jeong Y.-I., Park H.-K., Kang D.H., Oh J.-S., Lee S.-G., Lee H.C. (2015). Enzyme-responsive doxorubicin release from dendrimer nanoparticles for anticancer drug delivery. Int. J. Nanomed..

[25] Lin S.-C., Liao W.-L., Lee J.-C., Tsai S.-J. (2014). Hypoxia-regulated gene network in drug resistance and cancer progression. Exp. Biol. Med..

[26] Loscalzo J. (2016). Adaptions to Hypoxia and Redox Stress: Essential Concepts Confounded by Misleading Terminology. Circ. Res..

[27] Wang C., Wang J., Zhang X., Yu S., Wen D., Hu Q., Ye Y., Bomba H., Hu X., Liu Z. (2018). In situ formed reactive oxygen species–responsive scaffold with gemcitabine and checkpoint inhibitor for combination therapy. Sci. Transl. Med..

[28] Wang J., Zhang Y., Archibong E., Ligler F.S., Gu Z. (2017). Leveraging H2O2 Levels for Biomedical Applications. Adv. Biosyst..

[29] Ahmad Z., Lv S., Tang Z., Shah A., Chen X. (2016). Methoxy poly (ethylene glycol)-block-poly (glutamic acid)-graft-6-(2-nitroimidazole) hexyl amine nanoparticles for potential hypoxia-responsive delivery of doxorubicin. J. Biomater. Sci. Polym. Ed..

[30] Kulkarni P., Haldar M.K., You S., Choi Y., Mallik S. (2016). Hypoxia-Responsive Polymersomes for Drug Delivery to Hypoxic Pancreatic Cancer Cells. Biomacromolecules.

[31] Liu H.-M., Zhang Y.-F., Xie Y.-D., Cai Y.-F., Li B.-Y., Li W., Zeng L.-Y., Li Y.-L., Yu R.-T. (2017). Hypoxia-responsive ionizable liposome delivery siRNA for glioma therapy. Int. J. Nanomed..

[32] Yan Q., Guo X., Huang X., Meng X., Liu F., Dai P., Wang Z., Zhao Y. (2019). Gated Mesoporous Silica Nanocarriers for Hypoxia-Responsive Cargo Release. ACS Appl. Mater. Interfaces.

[33] Yatvin M.B., Weinstein J.N., Dennis W.H., Blumenthal R. (1978). Design of liposomes for enhanced local release of drugs by hyperthermia. Science.

[34] Smith B., Lyakhov I., Loomis K., Needle D., Baxa U., Yavlovich A., Capala J., Blumenthal R., Puri A. (2011). Hyperthermia-triggered intracellular delivery of anticancer agent to HER2+ cells by HER2-specific affibody (ZHER2-GS-Cys)-conjugated thermosensitive liposomes (HER2+ affisomes). J. Control. Release.

[35] Wu Q., Wang L., Yu H., Wang J., Chen Z. (2011). Organization of Glucose-Responsive Systems and Their Properties. Chem. Rev..

[36] Kost J., Langer R. (2001). Responsive polymeric delivery systems. Adv. Drug Deliv. Rev..

[37] Ghanem A., Ghaly A. (2004). Immobilization of glucose oxidase in chitosan gel beads. J. Appl. Polym. Sci..

[38] Kang S.I., Bae Y.H. (2003). A sulfonamide based glucose-responsive hydrogel with covalently immobilized glucose oxidase and catalase. J. Control. Release.

[39] Sharon N., Lis H. (1972). Lectins: Cell-Agglutinating and Sugar-Specific Proteins. Science.

[40] Pai C.M., Bae Y.H., Mack E.J., Wilson D.E., Kim S.W. (1992). Concanavalin a Microspheres for a Self-Regulating Insulin Delivery System. J. Pharm. Sci..

[41] Jamwal S., Ram B., Ranote S., Dharela R., Chauhan G.S. (2019). New glucose oxidase-immobilized stimuli-responsive dextran nanoparticles for insulin delivery. Int. J. Biol. Macromol..

[42] Tang W., Chen C. (2020). Hydrogel-Based Colloidal Photonic Crystal Devices for Glucose Sensing. Polymers.

[43] Zhao L., Huang Q., Liu Y., Wang Q., Wang L., Xiao S., Bi F., Ding J. (2017). Boronic acid as glucose-sensitive agent regulates drug delivery for diabetes treatment. Materials.

[44] Yan J., Fang H., Wang B. (2005). Boronolectins and fluorescent boronolectins: An examination of the detailed chemistry issues important for the design. Med. Res. Rev..

[45] Zhao W., Zhang H., He Q., Li Y., Gu J., Li L., Li H., Shi J. (2011). A glucose-responsive controlled release of insulin system based on enzyme multilayers-coated mesoporous silica particles. Chem. Commun..

[46] Xiang J., Tong X., Shi F., Yan Q., Yu B., Zhao Y. (2018). Near-infrared light-triggered drug release from UV-responsive diblock copolymer-coated upconversion nanoparticles with high monodispersity. J. Mater. Chem. B.

[47] Li H., Yang X., Zhou Z., Wang K., Li C., Qiao H., Oupicky D., Sun M. (2017). Near-infrared light-triggered drug release from a multiple lipid carrier complex using an all-in-one strategy. J. Control. Release.

[48] York A.G., Parekh S.H., Dalle Nogare D., Fischer R.S., Temprine K., Mione M., Chitnis A.B., Combs C.A., Shroff H. (2012). Resolution doubling in live, multicellular organisms via multifocal structured illumination microscopy. Nat. Methods.

[49] Jiang J., Tong X., Morris D., Zhao Y. (2006). Toward Photocontrolled Release Using Light-Dissociable Block Copolymer Micelles. Macromolecules.

[50] Babin J., Pelletier M., Lepage M., Allard J.-F., Morris D., Zhao Y. (2009). A New Two-Photon-Sensitive Block Copolymer Nanocarrier. Angew. Chem. Int. Ed..

[51] Chen Z., Yin J.-J., Zhou Y.-T., Zhang Y., Song L., Song M., Hu S., Gu N. (2012). Dual Enzyme-like Activities of Iron Oxide Nanoparticles and Their Implication for Diminishing Cytotoxicity. ACS Nano.

[52] Fang K., Song L., Gu Z., Yang F., Zhang Y., Gu N. (2015). Magnetic field activated drug release system based on magnetic PLGA microspheres for chemo-thermal therapy. Colloids Surf. B Biointerfaces.

[53] Yang F., Zhang X., Song L., Cui H., Myers J.N., Bai T., Zhou Y., Chen Z., Gu N. (2015). Controlled Drug Release and Hydrolysis Mechanism of Polymer–Magnetic Nanoparticle Composite. ACS Appl. Mater. Interfaces.

[54] Thirunavukkarasu G.K., Cherukula K., Lee H., Jeong Y.Y., Park I.-K., Lee J.Y. (2018). Magnetic field-inducible drug-eluting nanoparticles for image-guided thermo-chemotherapy. Biomaterials.

[55] Schleich N., Danhier F., Préat V. (2015). Iron oxide-loaded nanotheranostics: Major obstacles to in vivo studies and clinical translation. J. Control. Release.

[56] Paris J.L., Cabañas M.V., Manzano M., Vallet-Regí M. (2015). Polymer-Grafted Mesoporous Silica Nanoparticles as Ultrasound-Responsive Drug Carriers. ACS Nano.

[57] Mullick Chowdhury S., Lee T., Willmann J.K. (2017). Ultrasound-guided drug delivery in cancer. Ultrasonography.

[58] Yao J., Feng J., Chen J. (2016). External-stimuli responsive systems for cancer theranostic. Asian J. Pharm. Sci..

[59] El-Sherif D.M., Wheatley M.A. (2003). Development of a novel method for synthesis of a polymeric ultrasound contrast agent. J. Biomed. Mater. Res. Part A.

[60] El-Sherif D.M., Lathia J.D., Le N.T., Wheatley M.A. (2004). Ultrasound degradation of novel polymer contrast agents. J. Biomed. Mater. Res. Part A.

[61] Kruskal J., Goldberg S., Kane R. (2001). Novel in Vivo Use of Conventional Ultrasound to Guide and Enhance Molecular Delivery and Uptake into Solid Tumors. Radiology.

[62] Wang J., Xia Y., Liu H., Xia J., Qian M., Zhang L., Chen L., Chen Q. (2019). Poly(lactobionamidoethyl methacrylate)-based amphiphiles with ultrasound-labile components in manufacture of drug delivery nanoparticulates for augmented cytotoxic efficacy to hepatocellular carcinoma. J. Colloid Interface Sci..

[63] Jeon G., Yang S.Y., Byun J., Kim J.K. (2011). Electrically Actuatable Smart Nanoporous Membrane for Pulsatile Drug Release. Nano Lett..

[64] Servant A., Bussy C., Al-Jamal K., Kostarelos K. (2013). Design, engineering and structural integrity of electro-responsive carbon nanotube- based hydrogels for pulsatile drug release. J. Mater. Chem. B.

[65] Ge J., Neofytou E., Cahill T.J., Beygui R.E., Zare R.N. (2012). Drug Release from Electric-Field-Responsive Nanoparticles. ACS Nano.

[66] Hosseini-Nassab N., Samanta D., Abdolazimi Y., Annes J.P., Zare R.N. (2017). Electrically controlled release of insulin using polypyrrole nanoparticles. Nanoscale.

[67] Qu J., Liang Y., Shi M., Guo B., Gao Y., Yin Z. (2019). Biocompatible conductive hydrogels based on dextran and aniline trimer as electro-responsive drug delivery system for localized drug release. Int. J. Biol. Macromol..

[68] Majumder J., Taratula O., Minko T. (2019). Nanocarrier-based systems for targeted and site specific therapeutic delivery. Adv. Drug Deliv. Rev..

[69] Couvreur P., Vauthier C. (2006). Nanotechnology: Intelligent Design to Treat Complex Disease. Pharm. Res..

[70] Torchilin V.P. (2007). Targeted pharmaceutical nanocarriers for cancer therapy and imaging. Aaps J..

[71] Moghimi S.M., Hunter A.C., Murray J.C. (2005). Nanomedicine: Current status and future prospects. FASEB J..

[72] Shenoy D., Little S., Langer R., Amiji M. (2005). Poly(Ethylene Oxide)-Modified Poly(β-Amino Ester) Nanoparticles as a pH-Sensitive System for Tumor-Targeted Delivery of Hydrophobic Drugs: Part 2. In Vivo Distribution and Tumor Localization Studies. Pharm. Res..

[73] Shenoy D., Little S., Langer R., Amiji M. (2005). Poly(ethylene oxide)-Modified Poly(β-amino ester) Nanoparticles as a pH-Sensitive System for Tumor-Targeted Delivery of Hydrophobic Drugs. 1. In Vitro Evaluations. Mol. Pharm..

[74] Vaupel P., Kallinowski F., Okunieff P. (1989). Blood Flow, Oxygen and Nutrient Supply, and Metabolic Microenvironment of Human Tumors: A Review. Cancer Res..

[75] Meyer D.E., Shin B.C., Kong G.A., Dewhirst M.W., Chilkoti A. (2001). Drug targeting using thermally responsive polymers and local hyperthermia. J. Control. Release.

[76] Arruebo M., Fernández-Pacheco R., Ibarra M.R., Santamaría J. (2007). Magnetic nanoparticles for drug delivery. Nano Today.

[77] Ito A., Shinkai M., Honda H., Kobayashi T. (2005). Medical application of functionalized magnetic nanoparticles. J. Biosci. Bioeng..

[78] Gao Z.-G., Fain H.D., Rapoport N. (2005). Controlled and targeted tumor chemotherapy by micellar-encapsulated drug and ultrasound. J. Control. Release.

[79] Rapoport N. (2007). Physical stimuli-responsive polymeric micelles for anti-cancer drug delivery. Prog. Polym. Sci..

[80] Wagner E. (2007). Programmed drug delivery: Nanosystems for tumor targeting. Expert Opin. Biol. Ther..

[81] Sergeeva A., Kolonin M.G., Molldrem J.J., Pasqualini R., Arap W. (2006). Display technologies: Application for the discovery of drug and gene delivery agents. Adv. Drug Deliv. Rev..

[82] Schluesener H.J., Xianglin T. (2004). Selection of recombinant phages binding to pathological endothelial and tumor cells of rat glioblastoma by in-vivo display. J. Neurol. Sci..

[83] Bongartz T., Sutton A.J., Sweeting M.J., Buchan I., Matteson E.L., Montori V. (2006). Anti-TNF Antibody Therapy in Rheumatoid Arthritis and the Risk of Serious Infections and MalignanciesSystematic Review and Meta-analysis of Rare Harmful Effects in Randomized Controlled Trials. JAMA.

[84] Farokhzad O.C., Karp J.M., Langer R. (2006). Nanoparticle–aptamer bioconjugates for cancer targeting. Expert Opin. Drug Deliv..

[85] Kirpotin D.B., Drummond D.C., Shao Y., Shalaby M.R., Hong K., Nielsen U.B., Marks J.D., Benz C.C., Park J.W. (2006). Antibody Targeting of Long-Circulating Lipidic Nanoparticles Does Not Increase Tumor Localization but Does Increase Internalization in Animal Models. Cancer Res..

[86] Elbayoumi T.A., Pabba S., Roby A., Torchilin V.P. (2007). Antinucleosome Antibody-Modified Liposomes and Lipid-Core Micelles for Tumor-Targeted Delivery of Therapeutic and Diagnostic Agents. J. Liposome Res..

[87] Gupta B., Torchilin V.P. (2007). Monoclonal antibody 2C5-modified doxorubicin-loaded liposomes with significantly enhanced therapeutic activity against intracranial human brain U-87 MG tumor xenografts in nude mice. Cancer Immunol. Immunother..

[88] Blessing T., Kursa M., Holzhauser R., Kircheis R., Wagner E. (2001). Different Strategies for Formation of PEGylated EGF-Conjugated PEI/DNA Complexes for Targeted Gene Delivery. Bioconj. Chem..

[89] Leiden J.M. (1995). Gene Therapy—Promise, Pitfalls, and Prognosis. N. Engl. J. Med..

[90] Oishi M., Nagasaki Y. (2007). Synthesis, characterization, and biomedical applications of core–shell-type stimuli-responsive nanogels—Nanogel composed of poly[2-(N,N-diethylamino)ethyl methacrylate] core and PEG tethered chains. React. Funct. Polym..

[91] Ohtsuka N., Konno T., Miyauchi Y., Maeda H. (1987). Anticancer effects of arterial administration of the anticancer agent SMANCS with lipiodol on metastatic lymph nodes. Cancer.

[92] Allen T.M., Cullis P.R. (2004). Drug Delivery Systems: Entering the Mainstream. Science.

[93] Davis S.S. (1997). Biomédical applications of nanotechnology—implications for drug targeting and gene therapy. Trends Biotechnol..

[94] van Vlerken L.E., Duan Z., Seiden M.V., Amiji M.M. (2007). Modulation of Intracellular Ceramide Using Polymeric Nanoparticles to Overcome Multidrug Resistance in Cancer. Cancer Res..

[95] Kommareddy S., Tiwari S.B., Amiji M.M. (2005). Long-Circulating Polymeric Nanovectors for Tumor-Selective Gene Delivery. Technol. Cancer Res. Treat..

[96] Kaul G., Amiji M. (2002). Long-Circulating Poly(Ethylene Glycol)-Modified Gelatin Nanoparticles for Intracellular Delivery. Pharm. Res..

[97] Kaul G., Amiji M. (2005). Cellular Interactions and *In Vitro* DNA Transfection Studies with Poly(ethylene glycol-Modified Gelatin Nanoparticles. J. Pharm. Sci..

[98] Kommareddy S., Amiji M. (2007). Poly(ethylene glycol)-modified thiolated gelatin nanoparticles for glutathione-responsive intracellular DNA delivery. Nanomed. Nanotechnol. Biol. Med..

[99] Rasheed T., Bilal M., Abu-Thabit N.Y., Iqbal H.M.N., Makhlouf A.S.H., Abu-Thabit N.Y. (2018). 3—The smart chemistry of stimuli-responsive polymeric carriers for target drug delivery applications. Stimuli Responsive Polymeric Nanocarriers for Drug Delivery Applications, Volume 1.

[100] Rahdar A., Hajinezhad M.R., Nasri S., Beyzaei H., Barani M., Trant J.F. (2020). The synthesis of methotrexate-loaded F127 microemulsions and their in vivo toxicity in a rat model. J. Mol. Liq..

[101] Rahdar A., Sayyadi K., Sayyadi J., Yaghobi Z. (2019). Nano-gels: A versatile nano -carrier platform for drug delivery systems: A mini review. Nanomed. Res. J..

[102] Rahdar A., Taboada P., Hajinezhad M.R., Barani M., Beyzaei H. (2019). Effect of tocopherol on the properties of Pluronic F127 microemulsions: Physico-chemical characterization and in vivo toxicity. J. Mol. Liq..

[103] Rahdar A. (2019). Effect of tocopherol on Pluronic microemulsions: Turbidity studies and Dynamic light scattering and dynamic surface tension measurements. Nanomed. Res. J..

[104] Rahdar A., Kazemi S., Askari F. (2018). Pluronic as nano-carier for drug delivery systems. Nanomed. Res. J..

[105] Munnier E., Cohen-Jonathan S., Linassier C., Douziech-Eyrolles L., Marchais H., Soucé M., Hervé K., Dubois P., Chourpa I. (2008). Novel method of doxorubicin–SPION reversible association for magnetic drug targeting. Int. J. Pharm..

[106] Hashimoto Y., Tanaka M., Kishimoto H., Shiozawa H., Hasegawa K., Matsuyama K., Uchida T. (2002). Preparation, characterization and taste-masking properties of polyvinylacetal diethylaminoacetate microspheres containing trimebutine. J. Pharm. Pharmacol..

[107] Liang K., Richardson J.J., Ejima H., Such G.K., Cui J., Caruso F. (2014). Peptide-Tunable Drug Cytotoxicity via One-Step Assembled Polymer Nanoparticles. Adv. Mater..

[108] Wilson J.T., Keller S., Manganiello M.J., Cheng C., Lee C.-C., Opara C., Convertine A., Stayton P.S. (2013). pH-Responsive Nanoparticle Vaccines for Dual-Delivery of Antigens and Immunostimulatory Oligonucleotides. ACS Nano.

[109] Mao J., Kondu S., Ji H.-F., McShane M.J. (2006). Study of the near-neutral pH-sensitivity of chitosan/gelatin hydrogels by turbidimetry and microcantilever deflection. Biotechnol. Bioeng..

[110] Che Y., Li D., Liu Y., Ma Q., Tan Y., Yue Q., Meng F. (2016). Physically cross-linked pH-responsive chitosan-based hydrogels with enhanced mechanical performance for controlled drug delivery. Rsc Adv..

[111] Wang J., Wang F., Li X., Zhou Y., Wang H., Zhang Y. (2019). Uniform carboxymethyl chitosan-enveloped Pluronic F68/poly(lactic-co-glycolic acid) nano-vehicles for facilitated oral delivery of gefitinib, a poorly soluble antitumor compound. Colloids Surf. B Biointerfaces.

[112] Deen G.R., Loh X.J. (2018). Stimuli-Responsive Cationic Hydrogels in Drug Delivery Applications. Gels.

[113] Wu W., Liu J., Cao S., Tan H., Li J., Xu F., Zhang X. (2011). Drug release behaviors of a pH sensitive semi-interpenetrating polymer network hydrogel composed of poly(vinyl alcohol) and star poly[2-(dimethylamino)ethyl methacrylate]. Int. J. Pharm..

[114] Yang C., Liu W., Xiao J., Yuan C., Chen Y., Guo J., Yue H., Zhu D., Lin W., Tang S. (2020). pH-Sensitive Mixed Micelles Assembled from PDEAEMA-PPEGMA and PCL-PPEGMA for Doxorubicin Delivery: Experimental and DPD Simulations Study. Pharmaceutics.

[115] Radovic-Moreno A.F., Lu T.K., Puscasu V.A., Yoon C.J., Langer R., Farokhzad O.C. (2012). Surface charge-switching polymeric nanoparticles for bacterial cell wall-targeted delivery of antibiotics. ACS Nano.

[116] Chang C., He M., Zhou J., Zhang L. (2011). Swelling Behaviors of pH- and Salt-Responsive Cellulose-Based Hydrogels. Macromolecules.

[117] Zhao Y., Su H., Fang L., Tan T. (2005). Superabsorbent hydrogels from poly(aspartic acid) with salt-, temperature- and pH-responsiveness properties. Polymer.

[118] Yessine M.-A., Lafleur M., Meier C., Petereit H.-U., Leroux J.-C. (2003). Characterization of the membrane-destabilizing properties of different pH-sensitive methacrylic acid copolymers. Biochim. Biophys. Acta.

[119] Liu L., Yao W., Rao Y., Lu X., Gao J. (2017). pH-Responsive carriers for oral drug delivery: Challenges and opportunities of current platforms. Drug Deliv..

[120] Li G., Song S., Guo L., Ma S. (2008). Self-assembly of thermo- and pH-responsive poly(acrylic acid)-b-poly(N-isopropylacrylamide) micelles for drug delivery. J. Polym. Sci. Part A Polym. Chem..

[121] Wang X., Chen C., Huo D., Qian H., Ding Y., Hu Y., Jiang X. (2012). Synthesis of beta-cyclodextrin modified chitosan-poly(acrylic acid) nanoparticles and use as drug carriers. Carbohydr. Polym..

[122] Reis A.V., Moia T.A., Sitta D.L.A., Mauricio M.R., Tenório-Neto E.T., Guilherme M.R., Rubira A.F., Muniz E.C. (2016). Sustained release of potassium diclofenac from a pH-responsive hydrogel based on gum arabic conjugates into simulated intestinal fluid. J. Appl. Polym. Sci..

[123] Yu H., Zou Y., Wang Y., Huang X., Huang G., Sumer B.D., Boothman D.A., Gao J. (2011). Overcoming endosomal barrier by amphotericin B-loaded dual pH-responsive PDMA-b-PDPA micelleplexes for siRNA delivery. ACS Nano.

[124] de Oliveira K.A.L., Sitta D.L.A., Guilherme M.R., Muniz E.C., Rubira A.F. (2017). Design of pH-responsive albumin-alginate hydrogels for drug delivery. J. Control. Release.

[125] Kwon S.S., Kong B.J., Park S.N. (2015). Physicochemical properties of pH-sensitive hydrogels based on hydroxyethyl cellulose-hyaluronic acid and for applications as transdermal delivery systems for skin lesions. Eur. J. Pharm. Biopharm..

[126] Etrych T., Jelínková M., Říhová B., Ulbrich K. (2001). New HPMA copolymers containing doxorubicin bound via pH-sensitive linkage: Synthesis and preliminary in vitro and in vivo biological properties. J. Control. Release.

[127] Aryal S., Hu C.M., Zhang L. (2010). Polymer--cisplatin conjugate nanoparticles for acid-responsive drug delivery. ACS Nano.

[128] Wang C., Li P., Liu L., Pan H., Li H., Cai L., Ma Y. (2016). Self-adjuvanted nanovaccine for cancer immunotherapy: Role of lysosomal rupture-induced ROS in MHC class I antigen presentation. Biomaterials.

[129] Wang W., Yang H., Kong X., Ye Z., Yin Y., Zhang X., He G., Xu P., Zheng H. (2014). Hydrogen-bonding strategy for constructing pH-sensitive core–shell micelles with hydrophilic polymer as the shell and hydrophobic drug as the core. RSC Adv..

[130] Zhang X., Kang Y., Liu G.T., Li D.D., Zhang J.Y., Gu Z.P., Wu J. (2019). Poly(cystine-PCL) based pH/redox dual-responsive nanocarriers for enhanced tumor therapy. Biomater. Sci..

[131] Feng X., Li D., Han J., Zhuang X., Ding J. (2017). Schiff base bond-linked polysaccharide-doxorubicin conjugate for upregulated cancer therapy. Mater. Sci. Eng. C.

[132] Zhai Y., Zhou X., Jia L., Ma C., Song R., Deng Y., Hu X., Sun W. (2017). Acetal-Linked Paclitaxel Polymeric Prodrug Based on Functionalized mPEG-PCL Diblock Polymer for pH-Triggered Drug Delivery. Polymers.

[133] Shen W.C., Ryser H.J. (1981). cis-Aconityl spacer between daunomycin and macromolecular carriers: A model of pH-sensitive linkage releasing drug from a lysosomotropic conjugate. Biochem. Biophys. Res. Commun..

[134] Zhu S., Hong M., Zhang L., Tang G., Jiang Y., Pei Y. (2010). PEGylated PAMAM dendrimer-doxorubicin conjugates: In vitro evaluation and in vivo tumor accumulation. Pharm. Res..

[135] Lin C.J., Kuan C.H., Wang L.W., Wu H.C., Chen Y., Chang C.W., Huang R.Y., Wang T.W. (2016). Integrated self-assembling drug delivery system possessing dual responsive and active targeting for orthotopic ovarian cancer theranostics. Biomaterials.

[136] Saito G., Swanson J.A., Lee K.D. (2003). Drug delivery strategy utilizing conjugation via reversible disulfide linkages: Role and site of cellular reducing activities. Adv. Drug Deliv. Rev..

[137] Huo M., Yuan J., Tao L., Wei Y. (2014). Redox-responsive polymers for drug delivery: From molecular design to applications. Polym. Chem..

[138] Schafer F.Q., Buettner G.R. (2001). Redox environment of the cell as viewed through the redox state of the glutathione disulfide/glutathione couple. Free Radic. Biol. Med..

[139] Koo H., Jin G.W., Kang H., Lee Y., Nam K., Zhe Bai C., Park J.S. (2010). Biodegradable branched poly(ethylenimine sulfide) for gene delivery. Biomaterials.

[140] Song N., Liu W., Tu Q., Liu R., Zhang Y., Wang J. (2011). Preparation and in vitro properties of redox-responsive polymeric nanoparticles for paclitaxel delivery. Colloids Surf. B Biointerfaces.

[141] Sun P., Zhou D., Gan Z. (2011). Novel reduction-sensitive micelles for triggered intracellular drug release. J. Control. Release.

[142] Lee P.-Y., Tuan-Mu H.-Y., Hsiao L.-W., Hu J.-J., Jan J.-S. (2017). Nanogels comprising reduction-cleavable polymers for glutathione-induced intracellular curcumin delivery. J. Polym. Res..

[143] Wang Y.C., Wang F., Sun T.M., Wang J. (2011). Redox-responsive nanoparticles from the single disulfide bond-bridged block copolymer as drug carriers for overcoming multidrug resistance in cancer cells. Bioconj. Chem..

[144] Xia J., Du Y., Huang L., Chaurasiya B., Tu J., Webster T.J., Sun C. (2018). Redox-responsive micelles from disulfide bond-bridged hyaluronic acid-tocopherol succinate for the treatment of melanoma. Nanomedicine.

[145] Maiti C., Parida S., Kayal S., Maiti S., Mandal M., Dhara D. (2018). Redox-Responsive Core-Cross-Linked Block Copolymer Micelles for Overcoming Multidrug Resistance in Cancer Cells. ACS Appl. Mater. Interfaces.

[146] Huo M., Liu Y., Wang L., Yin T., Qin C., Xiao Y., Yin L., Liu J., Zhou J. (2016). Redox-Sensitive Micelles Based on O,N-Hydroxyethyl Chitosan-Octylamine Conjugates for Triggered Intracellular Delivery of Paclitaxel. Mol. Pharm..

[147] Abdullah Al N., Lee H., Lee Y.S., Lee K.D., Park S.Y. (2011). Development of disulfide core-crosslinked pluronic nanoparticles as an effective anticancer-drug-delivery system. Macromol. Biosci..

[148] Wen L., Hu Y., Meng T., Tan Y., Zhao M., Dai S., Yuan H., Hu F. (2019). Redox-responsive polymer inhibits macrophages uptake for effective intracellular gene delivery and enhanced cancer therapy. Colloids Surf. B Biointerfaces.

[149] Kommareddy S., Amiji M. (2005). Preparation and evaluation of thiol-modified gelatin nanoparticles for intracellular DNA delivery in response to glutathione. Bioconj. Chem..

[150] Hailemeskel B.Z., Hsu W.H., Addisu K.D., Andrgie A.T., Chou H.Y., Lai J.Y., Tsai H.C. (2019). Diselenide linkage containing triblock copolymer nanoparticles based on Bi(methoxyl poly(ethylene glycol))-poly(epsilon-carprolactone): Selective intracellular drug delivery in cancer cells. Mater. Sci. Eng. C.

[151] Birhan Y.S., Hailemeskel B.Z., Mekonnen T.W., Hanurry E.Y., Darge H.F., Andrgie A.T., Chou H.Y., Lai J.Y., Hsiue G.H., Tsai H.C. (2019). Fabrication of redox-responsive Bi(mPEG-PLGA)-Se2 micelles for doxorubicin delivery. Int. J. Pharm..

[152] Khandare J.J., Jayant S., Singh A., Chandna P., Wang Y., Vorsa N., Minko T. (2006). Dendrimer versus linear conjugate: Influence of polymeric architecture on the delivery and anticancer effect of paclitaxel. Bioconjugate Chem.

[153] Assali M., Shawahna R., Dayyeh S., Shareef M., Alhimony I.A. (2018). Dexamethasone-diclofenac loaded polylactide nanoparticles: Preparation, release and anti-inflammatory activity. Eur. J. Pharm. Sci..

[154] van Rijt S.H., Bolukbas D.A., Argyo C., Datz S., Lindner M., Eickelberg O., Konigshoff M., Bein T., Meiners S. (2015). Protease-mediated release of chemotherapeutics from mesoporous silica nanoparticles to ex vivo human and mouse lung tumors. ACS Nano.

[155] Zhu L., Perche F., Wang T., Torchilin V.P. (2014). Matrix metalloproteinase 2-sensitive multifunctional polymeric micelles for tumor-specific co-delivery of siRNA and hydrophobic drugs. Biomaterials.

[156] Wen J., Anderson S.M., Du J., Yan M., Wang J., Shen M., Lu Y., Segura T. (2011). Controlled protein delivery based on enzyme-responsive nanocapsules. Adv. Mater..

[157] Pan J., Li P.J., Wang Y., Chang L., Wan D., Wang H. (2018). Active targeted drug delivery of MMP-2 sensitive polymeric nanoparticles. Chem. Commun..

[158] Guo J., Sun H., Lei W., Tang Y., Hong S., Yang H., Tay F.R., Huang C. (2019). MMP-8-Responsive Polyethylene Glycol Hydrogel for Intraoral Drug Delivery. J. Dent. Res..

[159] Gianasi E., Buckley R.G., Latigo J., Wasil M., Duncan R. (2002). HPMA copolymers platinates containing dicarboxylato ligands. Preparation, characterisation and in vitro and in vivo evaluation. J. Drug Target..

[160] Zhang C., Pan D., Li J., Hu J., Bains A., Guys N., Zhu H., Li X., Luo K., Gong Q. (2017). Enzyme-responsive peptide dendrimer-gemcitabine conjugate as a controlled-release drug delivery vehicle with enhanced antitumor efficacy. Acta Biomater.

[161] Kern H.B., Srinivasan S., Convertine A.J., Hockenbery D., Press O.W., Stayton P.S. (2017). Enzyme-Cleavable Polymeric Micelles for the Intracellular Delivery of Proapoptotic Peptides. Mol. Pharm..

[162] Perche F., Biswas S., Wang T., Zhu L., Torchilin V.P. (2014). Hypoxia-targeted siRNA delivery. Angew. Chem. Int. Ed..

[163] Zhou Y., Maiti M., Sharma A., Won M., Yu L., Miao L.X., Shin J., Podder A., Bobba K.N., Han J. (2018). Azo-based small molecular hypoxia responsive theranostic for tumor-specific imaging and therapy. J. Control. Release.

[164] Son S., Rao N.V., Ko H., Shin S., Jeon J., Han H.S., Nguyen V.Q., Thambi T., Suh Y.D., Park J.H. (2018). Carboxymethyl dextran-based hypoxia-responsive nanoparticles for doxorubicin delivery. Int. J. Biol. Macromol..

[165] Kang L., Fan B., Sun P., Huang W., Jin M., Wang Q., Gao Z. (2016). An effective tumor-targeting strategy utilizing hypoxia-sensitive siRNA delivery system for improved anti-tumor outcome. Acta Biomater..

[166] Jeong B., Kibbey M.R., Birnbaum J.C., Won Y.-Y., Gutowska A. (2000). Thermogelling Biodegradable Polymers with Hydrophilic Backbones: PEG-g-PLGA. Macromolecules.

[167] Bae Y.H., Okano T., Kim S.W. (1991). “On-off” thermocontrol of solute transport. I. Temperature dependence of swelling of N-isopropylacrylamide networks modified with hydrophobic components in water. Pharm. Res..

[168] Zhang X.Z., Zhuo R.X., Cui J.Z., Zhang J.T. (2002). A novel thermo-responsive drug delivery system with positive controlled release. Int. J. Pharm..

[169] Bhattarai N., Matsen F.A., Zhang M. (2005). PEG-grafted chitosan as an injectable thermoreversible hydrogel. Macromol. Biosci..

[170] Gandhi S.S., Yan H., Kim C. (2014). Thermoresponsive Gelatin Nanogels. Acs Macro Lett..

[171] Cheng Y.H., Yang S.H., Su W.Y., Chen Y.C., Yang K.C., Cheng W.T., Wu S.C., Lin F.H. (2010). Thermosensitive chitosan-gelatin-glycerol phosphate hydrogels as a cell carrier for nucleus pulposus regeneration: An in vitro study. Tissue Eng. Part A.

[172] Kurisawa M., Yokoyama M., Okano T. (2000). Gene expression control by temperature with thermo-responsive polymeric gene carriers. J. Control. Release..

[173] Indulekha S., Arunkumar P., Bahadur D., Srivastava R. (2016). Thermoresponsive polymeric gel as an on-demand transdermal drug delivery system for pain management. Mater. Sci. Eng. C.

[174] Bouchemal K., Aka-Any-Grah A., Dereuddre-Bosquet N., Martin L., Lievin-Le-Moal V., Le Grand R., Nicolas V., Gibellini D., Lembo D., Pous C. (2015). Thermosensitive and mucoadhesive pluronic-hydroxypropylmethylcellulose hydrogel containing the mini-CD4 M48U1 is a promising efficient barrier against HIV diffusion through macaque cervicovaginal mucus. Antimicrob. Agents Chemother..

[175] Hacker M.C., Klouda L., Ma B.B., Kretlow J.D., Mikos A.G. (2008). Synthesis and characterization of injectable, thermally and chemically gelable, amphiphilic poly(N-isopropylacrylamide)-based macromers. Biomacromolecules.

[176] Hu J., Li H.-Y., Williams G.R., Yang H.-H., Tao L., Zhu L.-M. (2016). Electrospun Poly(N-isopropylacrylamide)/Ethyl Cellulose Nanofibers as Thermoresponsive Drug Delivery Systems. J. Pharm. Sci..

[177] Gu Z., Dang T.T., Ma M., Tang B.C., Cheng H., Jiang S., Dong Y., Zhang Y., Anderson D.G. (2013). Glucose-responsive microgels integrated with enzyme nanocapsules for closed-loop insulin delivery. ACS Nano.

[178] Hassan C.M., Doyle F.J., Peppas N.A. (1997). Dynamic Behavior of Glucose-Responsive Poly(methacrylic acid-g-ethylene glycol) Hydrogels. Macromolecules.

[179] Wang A., Fan W., Yang T., He S., Yang Y., Yu M., Fan L., Zhu Q., Guo S., Zhu C. (2020). Liver-Target and Glucose-Responsive Polymersomes toward Mimicking Endogenous Insulin Secretion with Improved Hepatic Glucose Utilization. Adv. Funct. Mater..

[180] VandenBerg M.A., Webber M.J. (2019). Biologically Inspired and Chemically Derived Methods for Glucose-Responsive Insulin Therapy. Adv. Healthc. Mater..

[181] Miyata T., Jikihara A., Nakamae K., Hoffman A.S. (1996). Preparation of poly(2-glucosyloxyethyl methacrylate)-concanavalin A complex hydrogel and its glucose-sensitivity. Macromol. Chem. Phys..

[182] Tanna S., Joan Taylor M., Sahota T.S., Sawicka K. (2006). Glucose-responsive UV polymerised dextran-concanavalin A acrylic derivatised mixtures for closed-loop insulin delivery. Biomaterials.

[183] Chang R., Li M., Ge S., Yang J., Sun Q., Xiong L. (2018). Glucose-responsive biopolymer nanoparticles prepared by co-assembly of concanavalin A and amylopectin for insulin delivery. Ind. Crop. Prod..

[184] Lee K., Bae K.H., Lee Y., Lee S.H., Ahn C.H., Park T.G. (2010). Pluronic/polyethylenimine shell crosslinked nanocapsules with embedded magnetite nanocrystals for magnetically triggered delivery of siRNA. Macromol. Biosci..

[185] Mohapatra A., Harris M.A., LeVine D., Ghimire M., Jennings J.A., Morshed B.I., Haggard W.O., Bumgardner J.D., Mishra S.R., Fujiwara T. (2018). Magnetic stimulus responsive vancomycin drug delivery system based on chitosan microbeads embedded with magnetic nanoparticles. J. Biomed. Mater. Res. Part B Appl. Biomater..

[186] Liu H., Liu J., Xie X., Li X. (2020). Development of photo-magnetic drug delivery system by facile-designed dual stimuli-responsive modified biopolymeric chitosan capped nano-vesicle to improve efficiency in the anesthetic effect and its biological investigations. J. Photochem. Photobiol. B Biol..

[187] Ghamkhari A., Ghorbani M., Aghbolaghi S. (2018). A perfect stimuli-responsive magnetic nanocomposite for intracellular delivery of doxorubicin. Artif. Cells Nanomed. Biotechnol..

[188] Silva-Freitas E.L., Pontes T.R.F., Araujo-Neto R.P., Damasceno I.H.M., Silva K.L., Carvalho J.F., Medeiros A.C., Silva R.B., Silva A.K.A., Morales M.A. (2017). Design of Magnetic Polymeric Particles as a Stimulus-Responsive System for Gastric Antimicrobial Therapy. AAPS PharmSciTech.

[189] Yang G., Liu J., Wu Y., Feng L., Liu Z. (2016). Near-infrared-light responsive nanoscale drug delivery systems for cancer treatment. Coord. Chem. Rev..

[190] Cabane E., Malinova V., Menon S., Palivan C.G., Meier W. (2011). Photoresponsive polymersomes as smart, triggerable nanocarriers. Soft Matter.

[191] Fischer W., Quadir M.A., Barnard A., Smith D.K., Haag R. (2011). Controlled release of DNA from photoresponsive hyperbranched polyglycerols with oligoamine shells. Macromol. Biosci..

[192] Deveci G., Kahveci M.U. (2018). One-pot one-step synthesis of a photo-cleavable cross-linker via Passerini reaction for fabrication of responsive polymeric particles. Polym. Bull..

[193] Jin Q., Mitschang F., Agarwal S. (2011). Biocompatible drug delivery system for photo-triggered controlled release of 5-Fluorouracil. Biomacromolecules.

[194] Pauly A.C., Scholler K., Baumann L., Rossi R.M., Dustmann K., Ziener U., de Courten D., Wolf M., Boesel L.F., Scherer L.J. (2015). ATRP-based synthesis and characterization of light-responsive coatings for transdermal delivery systems. Sci. Technol. Adv. Mater..

[195] Son S., Shin E., Kim B.S. (2014). Light-responsive micelles of spiropyran initiated hyperbranched polyglycerol for smart drug delivery. Biomacromolecules.

[196] Razavi B., Abdollahi A., Roghani-Mamaqani H., Salami-Kalajahi M. (2020). Light-, temperature-, and pH-responsive micellar assemblies of spiropyran-initiated amphiphilic block copolymers: Kinetics of photochromism, responsiveness, and smart drug delivery. Mater. Sci. Eng. C.

[197] Wang Y., Han P., Xu H., Wang Z., Zhang X., Kabanov A.V. (2010). Photocontrolled self-assembly and disassembly of block ionomer complex vesicles: A facile approach toward supramolecular polymer nanocontainers. Langmuir.

[198] Peng K., Tomatsu I., Kros A. (2010). Light controlled protein release from a supramolecular hydrogel. Chem. Commun..

[199] Jin H., Tan H., Zhao L., Sun W., Zhu L., Sun Y., Hao H., Xing H., Liu L., Qu X. (2012). Ultrasound-triggered thrombolysis using urokinase-loaded nanogels. Int. J. Pharm..

[200] Wu D., Wan M. (2008). A novel fluoride anion modified gelatin nanogel system for ultrasound-triggered drug release. J. Pharm. Pharm. Sci..

[201] Husseini G.A., Abdel-Jabbar N.M., Mjalli F.S., Pitt W.G. (2011). Optimizing the use of ultrasound to deliver chemotherapeutic agents to cancer cells from polymeric micelles. J. Frankl. Inst..

[202] Wu P., Jia Y., Qu F., Sun Y., Wang P., Zhang K., Xu C., Liu Q., Wang X. (2017). Ultrasound-Responsive Polymeric Micelles for Sonoporation-Assisted Site-Specific Therapeutic Action. ACS Appl. Mater. Interfaces.

[203] Rapoport N.Y., Kennedy A.M., Shea J.E., Scaife C.L., Nam K.H. (2009). Controlled and targeted tumor chemotherapy by ultrasound-activated nanoemulsions/microbubbles. J. Control. Release.

[204] Zhou X., Guo L., Shi D., Duan S., Li J. (2019). Biocompatible Chitosan Nanobubbles for Ultrasound-Mediated Targeted Delivery of Doxorubicin. Nanoscale Res. Lett..

[205] Yin T., Wang P., Li J., Wang Y., Zheng B., Zheng R., Cheng D., Shuai X. (2014). Tumor-penetrating codelivery of siRNA and paclitaxel with ultrasound-responsive nanobubbles hetero-assembled from polymeric micelles and liposomes. Biomaterials.

[206] Yang H., Deng L., Li T., Shen X., Yan J., Zuo L., Wu C., Liu Y. (2015). Multifunctional PLGA Nanobubbles as Theranostic Agents: Combining Doxorubicin and P-gp siRNA Co-Delivery Into Human Breast Cancer Cells and Ultrasound Cellular Imaging. J. Biomed. Nanotechnol..

[207] Nguyen K., Pan H.Y., Haworth K., Mahoney E., Mercado-Shekhar K.P., Lin C.Y., Zhang Z., Park Y.C. (2019). Multiple-Exposure Drug Release from Stable Nanodroplets by High-Intensity Focused Ultrasound for a Potential Degenerative Disc Disease Treatment. Ultrasound Med. Biol..

[208] Yang Z., Luo X., Zhang X., Liu J., Jiang Q. (2013). Targeted delivery of 10-hydroxycamptothecin to human breast cancers by cyclic RGD-modified lipid-polymer hybrid nanoparticles. Biomed. Mater..

[209] Liu J., Xu F., Huang J., Xu J., Liu Y., Yao Y., Ao M., Li A., Hao L., Cao Y. (2018). Low-intensity focused ultrasound (LIFU)-activated nanodroplets as a theranostic agent for noninvasive cancer molecular imaging and drug delivery. Biomater. Sci..

[210] Cao Y., Chen Y., Yu T., Guo Y., Liu F., Yao Y., Li P., Wang D., Wang Z., Chen Y. (2018). Drug Release from Phase-Changeable Nanodroplets Triggered by Low-Intensity Focused Ultrasound. Theranostics.

[211] Gao J., Yu B., Li C., Xu M., Cao Z., Xie X., Wang W., Liu J. (2019). Ultrasound triggered phase-change nanodroplets for doxorubicin prodrug delivery and ultrasound diagnosis: An in vitro study. Colloids Surf. B Biointerfaces.

[212] Chen J., Yu M., Guo B., Ma P.X., Yin Z. (2018). Conductive nanofibrous composite scaffolds based on in-situ formed polyaniline nanoparticle and polylactide for bone regeneration. J. Colloid Interface Sci..

[213] Zhao P., Liu H., Deng H., Xiao L., Qin C., Du Y., Shi X. (2014). A study of chitosan hydrogel with embedded mesoporous silica nanoparticles loaded by ibuprofen as a dual stimuli-responsive drug release system for surface coating of titanium implants. Colloids Surf. B Biointerfaces.

[214] Ying X., Wang Y., Liang J., Yue J., Xu C., Lu L., Xu Z., Gao J., Du Y., Chen Z. (2014). Angiopep-conjugated electro-responsive hydrogel nanoparticles: Therapeutic potential for epilepsy. Angew. Chem..

[215] Abelha T.F., Dreiss C.A., Green M., Dailey L.A. (2020). Conjugated polymers as nanoparticle probes for fluorescence and photoacoustic imaging. J. Mater. Chem. B.

[216] Chatterjee S., Hui C.-L. (2019). Review of Stimuli-Responsive Polymers in Drug Delivery and Textile Application. Molecules.

[217] Xu Y. (2018). Synthesis Of Small-Molecule Fluorescent Probe And Polymers For Bioapplication: Bioimaging And Enzyme Stabilization. MSc thesis.

[218] Etrych T., Lucas H., Janoušková O., Chytil P., Mueller T., Mäder K. (2016). Fluorescence optical imaging in anticancer drug delivery. J. Control. Release.

[219] Crich S.G., Terreno E., Aime S. (2017). Nano-sized and other improved reporters for magnetic resonance imaging of angiogenesis. Adv. Drug Deliv. Rev..

[220] Zhu W., Yang Y., Jin Q., Chao Y., Tian L., Liu J., Dong Z., Liu Z. (2019). Two-dimensional metal-organic-framework as a unique theranostic nano-platform for nuclear imaging and chemo-photodynamic cancer therapy. Nano Res..

[221] Luke G.P., Hannah A.S., Emelianov S.Y. (2016). Super-resolution ultrasound imaging in vivo with transient laser-activated nanodroplets. Nano Lett..

[222] Li J., Pu K. (2019). Development of organic semiconducting materials for deep-tissue optical imaging, phototherapy and photoactivation. Chem. Soc. Rev..

[223] Carr J.A., Franke D., Caram J.R., Perkinson C.F., Saif M., Askoxylakis V., Datta M., Fukumura D., Jain R.K., Bawendi M.G. (2018). Shortwave infrared fluorescence imaging with the clinically approved near-infrared dye indocyanine green. Proc. Natl. Acad. Sci. USA.

[224] Nagamani N., Lakshmanan S., Govindaraj D., Ramamoorthy C., Ramalakshmi N., Antony S.A. (2019). Selective and efficient detection of picric acid among other nitroaromatics by NIR fluorescent cyanine chemosensors. Spectrochim. Acta Part A Mol. Biomol. Spectrosc..

[225] Wang T.-C., Cochet F., Facchini F.A., Zaffaroni L., Serba C., Pascal S., Andraud C., Sala A., Di Lorenzo F., Maury O. (2019). Synthesis of the New Cyanine-Labeled Bacterial Lipooligosaccharides for Intracellular Imaging and in Vitro Microscopy Studies. Bioconj. Chem..

[226] Mahmoud A.M., De Jongh P.A., Briere S., Chen M., Nowell C.J., Johnston A.P., Davis T.P., Haddleton D.M., Kempe K. (2019). Carboxylated Cy5-Labeled Comb Polymers Passively Diffuse the Cell Membrane and Target Mitochondria. ACS Appl. Mater. Interfaces.

[227] Mu X., Lu Y., Wu F., Wei Y., Ma H., Zhao Y., Sun J., Liu S., Zhou X., Li Z. (2020). Supramolecular Nanodiscs Self-Assembled from Non-Ionic Heptamethine Cyanine for Imaging-Guided Cancer Photothermal Therapy. Adv. Mater..

[228] Shao C., Xiao F., Guo H., Yu J., Jin D., Wu C., Xi L., Tian L. (2019). Utilizing Polymer Micelle to Control Dye J-aggregation and Enhance Its Theranostic Capability. iScience.

[229] Xiaoyang L., Hao-Ran J., Ya-Xuan Z., Ge G., Yao-Wen J., Xiaotong C., Ke-Fei X., Xin-Wang Y., Fu-Gen W. (2020). Mitochondrion-and nucleus-acting polymeric nanoagents for chemo-photothermal combination therapy. Sci. China Mater..

[230] Yang X., An J., Luo Z., Yang R., Yan S., Liu D.-E., Fu H., Gao H. (2020). A cyanine-based polymeric nanoplatform with microenvironment-driven cascaded responsiveness for imaging-guided chemo-photothermal combination anticancer therapy. J. Mater. Chem. B.

[231] Sun C., Li B., Zhao M., Wang S., Lei Z., Lu L., Zhang H., Feng L., Dou C., Yin D. (2019). J-Aggregates of Cyanine Dye for NIR-II in Vivo Dynamic Vascular Imaging beyond 1500 nm. J. Am. Chem. Soc..

[232] Martínez R., Polo E., Barbosa S., Taboada P., del Pino P., Pelaz B. (2020). 808 nm-activable core@ multishell upconverting nanoparticles with enhanced stability for efficient photodynamic therapy. J. Nanobiotechnol..

[233] Liu J.-B., Merton D.A., Forsberg F., Goldberg B.B. (2019). Contrast-enhanced ultrasound imaging. Diagnostic Ultrasound.

[234] Prabhakar A., Banerjee R. (2019). Nanobubble Liposome Complexes for Diagnostic Imaging and Ultrasound-Triggered Drug Delivery in Cancers: A Theranostic Approach. ACS Omega.

[235] Shang M., Wang K., Guo L., Duan S., Lu Z., Li J. (2019). Development of novel ST68/PLA-PEG stabilized ultrasound nanobubbles for potential tumor imaging and theranostic. Ultrasonics.

[236] Zhang X., Zheng Y., Wang Z., Huang S., Chen Y., Jiang W., Zhang H., Ding M., Li Q., Xiao X. (2014). Methotrexate-loaded PLGA nanobubbles for ultrasound imaging and synergistic targeted therapy of residual tumor during HIFU ablation. Biomaterials.

[237] Lee J.Y., Carugo D., Crake C., Owen J., de Saint Victor M., Seth A., Coussios C., Stride E. (2015). Nanoparticle-loaded protein–polymer nanodroplets for improved stability and conversion efficiency in ultrasound imaging and drug delivery. Adv. Mater..

[238] Vijayan V.M., Muthu J. (2017). Polymeric nanocarriers for cancer theranostics. Polym. Adv. Technol..

[239] Mi P., Wang F., Nishiyama N., Cabral H. (2017). Molecular cancer imaging with polymeric nanoassemblies: From tumor detection to theranostics. Macromol. Biosci..

[240] Luk B.T., Zhang L. (2014). Current advances in polymer-based nanotheranostics for cancer treatment and diagnosis. ACS Appl. Mater. Interfaces.

[241] Hu H. (2020). Recent Advances of Bioresponsive Nano-Sized Contrast Agents for Ultra-High-Field Magnetic Resonance Imaging. Front. Chem..

[242] Munkhbat O., Canakci M., Zheng S., Hu W., Osborne B., Bogdanov A.A., Thayumanavan S. (2018). 19F MRI of polymer nanogels aided by improved segmental mobility of embedded fluorine moieties. Biomacromolecules.

[243] Bain J., Legge C.J., Beattie D.L., Sahota A., Dirks C., Lovett J.R., Staniland S.S. (2019). A biomimetic magnetosome: Formation of iron oxide within carboxylic acid terminated polymersomes. Nanoscale.

[244] Aouidat F., Boumati S., Khan M., Tielens F., Doan B.-T., Spadavecchia J. (2019). Design and Synthesis of Gold-Gadolinium-Core-Shell Nanoparticles as contrast agent: A Smart Way to Future Nanomaterials for Nanomedicine Applications. Int. J. Nanomed..

[245] Valluru K.S., Willmann J.K. (2016). Clinical photoacoustic imaging of cancer. Ultrasonography.

[246] Zhou W., Zheng S., Schultz J.W., Rath N.P., Mirica L.M. (2016). Aromatic cyanoalkylation through double C–H activation mediated by Ni (III). J. Am. Chem. Soc..

[247] Zhang J., Yang C., Zhang R., Chen R., Zhang Z., Zhang W., Peng S.H., Chen X., Liu G., Hsu C.S. (2017). Biocompatible D–A Semiconducting Polymer Nanoparticle with Light-Harvesting Unit for Highly Effective Photoacoustic Imaging Guided Photothermal Therapy. Adv. Funct. Mater..

[248] Lyu Y., Fang Y., Miao Q., Zhen X., Ding D., Pu K. (2016). Intraparticle molecular orbital engineering of semiconducting polymer nanoparticles as amplified theranostics for in vivo photoacoustic imaging and photothermal therapy. ACS Nano.

[249] Liu Y., Ai K., Lu L. (2012). Nanoparticulate X-ray computed tomography contrast agents: From design validation to in vivo applications. Acc. Chem. Res..

[250] Anton N., Vandamme T.F. (2014). Nanotechnology for computed tomography: A real potential recently disclosed. Pharm. Res..

[251] Dong Y.C., Hajfathalian M., Maidment P.S., Hsu J.C., Naha P.C., Si-Mohamed S., Breuilly M., Kim J., Chhour P., Douek P. (2019). Effect of gold nanoparticle size on their properties as contrast agents for computed tomography. Sci. Rep..

[252] Jang J.D., Jeon S.-W., Yoon Y.-J., Bang J., Han Y.S., Kim T.-H. (2019). Self-assembly of gold nanoparticles in a block copolymer aggregate template driven by hydrophobic interactions. Polym. Chem..

[253] Shapoval O., Kaman O., Hromádková J., Vavřík D., Jirák D., Machová D., Parnica J., Horák D. (2019). Multimodal PSSMA-Functionalized GdF_3_: Eu^3+^ (Tb^3+^) Nanoparticles for Luminescence Imaging, MRI, and X-Ray Computed Tomography. ChemPlusChem.

[254] Pant K., Sedláček O., Nadar R.A., Hrubý M., Stephan H. (2017). Radiolabelled polymeric materials for imaging and treatment of cancer: Quo vadis?. Adv. Healthc. Mater..

[255] Di Mauro P.P., Goómez-Vallejo V., Baz Maldonado Z., Llop Roig J., Borroós S. (2015). Novel 18F labeling strategy for polyester-based NPs for in vivo PET-CT imaging. Bioconj. Chem..

[256] Sun J., Sun L., Li J., Xu J., Wan Z., Ouyang Z., Liang L., Li S., Zeng D. (2018). A multi-functional polymeric carrier for simultaneous positron emission tomography imaging and combination therapy. Acta Biomater..

[257] Sun N., Zhao L., Zhu J., Li Y., Song N., Xing Y., Qiao W., Huang H., Zhao J. (2019). 131I-labeled polyethylenimine-entrapped gold nanoparticles for targeted tumor SPECT/CT imaging and radionuclide therapy. Int. J. Nanomed..

[258] Le Goas M., Paquet M., Paquirissamy A., Guglielmi J., Compin C., Thariat J., Vassaux G., Geertsen V., Humbert O., Renault J.-P. (2019). Improving 131I radioiodine therapy by hybrid polymer-grafted gold nanoparticles. Int. J. Nanomed..

[259] Yang L., Zhang C., Liu J., Huang F., Zhang Y., Liang X.J., Liu J. (2020). ICG-Conjugated and 125I-Labeled Polymeric Micelles with High Biosafety for Multimodality Imaging-Guided Photothermal Therapy of Tumors. Adv. Healthc. Mater..

[260] Song G., Zheng X., Wang Y., Xia X., Chu S., Rao J. (2019). A magneto-optical nanoplatform for multimodality imaging of tumors in mice. ACS Nano.

[261] Hu X., Tang Y., Hu Y., Lu F., Lu X., Wang Y., Li J., Li Y., Ji Y., Wang W. (2019). Gadolinium-chelated conjugated polymer-based nanotheranostics for photoacoustic/magnetic resonance/NIR-II fluorescence imaging-guided cancer photothermal therapy. Theranostics.

[262] Zhang M., Liu J., Wang G. (2019). Highly Biocompatible Nanoparticles of Au@ Fluorescent Polymers as Novel Contrast Agent for In Vivo Bimodality NIR Fluorescence/CT Imaging. Contrast Media Mol. Imaging.

[263] Elsabahy M., Heo G.S., Lim S.-M., Sun G., Wooley K.L. (2015). Polymeric nanostructures for imaging and therapy. Chem. Rev..

[264] Krasia-Christoforou T., Georgiou T.K. (2013). Polymeric theranostics: Using polymer-based systems for simultaneous imaging and therapy. J. Mater. Chem. B.

[265] Jiang Y., Wang Y., Li Q., Chen Y., Chu W. (2020). Natural polymer-based stimuli-responsive hydrogels. Curr. Med. Chem..

[266] Tong X., Pan W., Su T., Zhang M., Dong W., Qi X. (2020). Recent advances in natural polymer-based drug delivery systems. React. Funct. Polym..

[267] Chen F., Ehlerding E.B., Cai W. (2014). Theranostic nanoparticles. J. Nucl. Med..

[268] Allen T.M. (2002). Ligand-targeted therapeutics in anticancer therapy. Nat. Rev. Cancer.

[269] Virgolini I., Traub T., Novotny C., Leimer M., Fuger B., Li S., Patri P., Pangerl T., Angelberger P., Raderer M. (2002). Experience with indium-111 and yttrium-90-labeled somatostatin analogs. Curr. Pharm. Des..

[270] Fan Y., Zhang J., Shi M., Li D., Lu C., Cao X., Peng C., Mignani S., Majoral J.-P., Shi X. (2019). Poly (amidoamine) dendrimer-coordinated copper (II) complexes as a theranostic nanoplatform for the radiotherapy-enhanced magnetic resonance imaging and chemotherapy of tumors and tumor metastasis. Nano Lett..

[271] Won Y.-W., Patel A.N., Bull D.A. (2014). Cell surface engineering to enhance mesenchymal stem cell migration toward an SDF-1 gradient. Biomaterials.

[272] Mei L., Rao J., Liu Y., Li M., Zhang Z., He Q. (2018). Effective treatment of the primary tumor and lymph node metastasis by polymeric micelles with variable particle sizes. J. Control. Release.

[273] Zhang H., Mi P. (2019). Polymeric Micelles for Tumor Theranostics. Theranostic Bionanomaterials.

[274] Cabral H., Miyata K., Osada K., Kataoka K. (2018). Block copolymer micelles in nanomedicine applications. Chem. Rev..

[275] Shih Y.-H., Luo T.-Y., Chiang P.-F., Yao C.-J., Lin W.-J., Peng C.-L., Shieh M.-J. (2017). EGFR-targeted micelles containing near-infrared dye for enhanced photothermal therapy in colorectal cancer. J. Control. Release.

[276] Bukchin A., Pascual-Pasto G., Cuadrado-Vilanova M., Castillo-Ecija H., Monterrubio C., Olaciregui N.G., Vila-Ubach M., Ordeix L., Mora J., Carcaboso A.M. (2018). Glucosylated nanomicelles target glucose-avid pediatric patient-derived sarcomas. J. Control. Release.

[277] Tang B., Zaro J.L., Shen Y., Chen Q., Yu Y., Sun P., Wang Y., Shen W.-C., Tu J., Sun C. (2018). Acid-sensitive hybrid polymeric micelles containing a reversibly activatable cell-penetrating peptide for tumor-specific cytoplasm targeting. J. Control. Release.

[278] Chen C., Zhao J., Gao M., Meng X., Fan A., Wang Z., Zhao Y. (2018). Photo-triggered micelles: Simultaneous activation and release of microtubule inhibitors for on-demand chemotherapy. Biomater. Sci..

[279] Pourjavadi A., Bagherifard M., Doroudian M. (2020). Synthesis of micelles based on chitosan functionalized with gold nanorods as a light sensitive drug delivery vehicle. Int. J. Biol. Macromol..

[280] Zhu L., Li P., Gao D., Liu J., Liu Y., Sun C., Xu M., Chen X., Sheng Z., Wang R. (2019). pH-sensitive loaded retinal/indocyanine green micelles as an “all-in-one” theranostic agent for multi-modal imaging in vivo guided cellular senescence-photothermal synergistic therapy. Chem. Commun..

[281] Yang H., Wang N., Mo L., Wu M., Yang R., Xu X., Huang Y., Lin J., Zhang L.-M., Jiang X. (2019). Reduction sensitive hyaluronan-SS-poly (ε-caprolactone) block copolymers as theranostic nanocarriers for tumor diagnosis and treatment. Mater. Sci. Eng. C.

[282] Luk B.T., Fang R.H., Zhang L. (2012). Lipid-and polymer-based nanostructures for cancer theranostics. Theranostics.

[283] Lee W., Im H.-J. (2019). Theranostics Based on Liposome: Looking Back and Forward. Nucl. Med. Mol. Imaging.

[284] Malam Y., Loizidou M., Seifalian A.M. (2009). Liposomes and nanoparticles: Nanosized vehicles for drug delivery in cancer. Trends Pharmacol. Sci..

[285] Immordino M.L., Dosio F., Cattel L. (2006). Stealth liposomes: Review of the basic science, rationale, and clinical applications, existing and potential. Int. J. Nanomed..

[286] Bovier P.A. (2008). Epaxal®: A virosomal vaccine to prevent hepatitis A infection. Expert Rev. Vaccines.

[287] Gardikis K., Tsimplouli C., Dimas K., Micha-Screttas M., Demetzos C. (2010). New chimeric advanced Drug Delivery nano Systems (chi-aDDnSs) as doxorubicin carriers. Int. J. Pharm..

[288] Silverman J.A., Deitcher S.R. (2013). Marqibo®(vincristine sulfate liposome injection) improves the pharmacokinetics and pharmacodynamics of vincristine. Cancer Chemother. Pharmacol..

[289] Bulbake U., Doppalapudi S., Kommineni N., Khan W. (2017). Liposomal formulations in clinical use: An updated review. Pharmaceutics.

[290] Zhao Y., Ren W., Zhong T., Zhang S., Huang D., Guo Y., Yao X., Wang C., Zhang W.-Q., Zhang X. (2016). Tumor-specific pH-responsive peptide-modified pH-sensitive liposomes containing doxorubicin for enhancing glioma targeting and anti-tumor activity. J. Control. Release.

[291] Mansoori B., Mohammadi A., Abedi-Gaballu F., Abbaspour S., Ghasabi M., Yekta R., Shirjang S., Dehghan G., Hamblin M.R., Baradaran B. (2020). Hyaluronic acid-decorated liposomal nanoparticles for targeted delivery of 5-fluorouracil into HT-29 colorectal cancer cells. J. Cell. Physiol..

[292] Fu M., Tang W., Liu J.-J., Gong X.-Q., Kong L., Yao X.-M., Jing M., Cai F.-Y., Li X.-T., Ju R.-J. (2020). Combination of targeted daunorubicin liposomes and targeted emodin liposomes for treatment of invasive breast cancer. J. Drug Target..

[293] Cheng Y., Wang J., Rao T., He X., Xu T. (2008). Pharmaceutical applications of dendrimers: Promising nanocarriers for drug delivery. Front. Biosci..

[294] Šebestík J., Reiniš M., Ježek J. (2012). Synthesis of dendrimers: Convergent and divergent approaches. Biomedical Applications of Peptide-, Glyco-and Glycopeptide Dendrimers, and Analogous Dendrimeric Structures.

[295] Grayson S.M., Frechet J.M. (2001). Convergent dendrons and dendrimers: From synthesis to applications. Chem. Rev..

[296] Cheng Y., Xu Z., Ma M., Xu T. (2008). Dendrimers as drug carriers: Applications in different routes of drug administration. J. Pharm. Sci..

[297] Zhu Y., Liu C., Pang Z. (2019). Dendrimer-Based Drug Delivery Systems for Brain Targeting. Biomolecules.

[298] Zhang H., Ma Y., Xie Y., An Y., Huang Y., Zhu Z., Yang C.J. (2015). A controllable aptamer-based self-assembled DNA dendrimer for high affinity targeting, bioimaging and drug delivery. Sci. Rep..

[299] Li Y., Li Y., Zhang X., Xu X., Zhang Z., Hu C., He Y., Gu Z. (2016). Supramolecular PEGylated dendritic systems as pH/redox dual-responsive theranostic nanoplatforms for platinum drug delivery and NIR imaging. Theranostics.

[300] Jędrzak A., Grześkowiak B.F., Coy E., Wojnarowicz J., Szutkowski K., Jurga S., Jesionowski T., Mrówczyński R. (2019). Dendrimer based theranostic nanostructures for combined chemo-and photothermal therapy of liver cancer cells in vitro. Colloids Surf. B Biointerfaces.

[301] Taratula O., Schumann C., Duong T., Taylor K.L., Taratula O. (2015). Dendrimer-encapsulated naphthalocyanine as a single agent-based theranostic nanoplatform for near-infrared fluorescence imaging and combinatorial anticancer phototherapy. Nanoscale.

[302] Sultana F., Manirujjaman M., Imran-Ul-Haque M.A., Sharmin S. (2013). An overview of nanogel drug delivery system. J. Appl. Pharm. Sci..

[303] Oh J.K., Drumright R., Siegwart D.J., Matyjaszewski K. (2008). The development of microgels/nanogels for drug delivery applications. Prog. Polym. Sci..

[304] Zha L., Banik B., Alexis F. (2011). Stimulus responsive nanogels for drug delivery. Soft Matter.

[305] Cho M.H., Li Y., Lo P.-C., Lee H., Choi Y. (2020). Fucoidan-Based Theranostic Nanogel for Enhancing Imaging and Photodynamic Therapy of Cancer. Nano-Micro Lett..

[306] Shi X., Zou Y., Li D., Wang Y., Ouyang Z., Peng Y., Tomás H., Xia J., Rodrigues J.M., Shen M. (2020). Polyethylenimine Nanogels Incorporated with Ultrasmall Iron Oxide Nanoparticles and Doxorubicin for MR Imaging-Guided Chemotherapy of Tumors. Bioconj. Chem..

[307] Gao F., Wu X., Wu D., Yu J., Yao J., Qi Q., Cao Z., Cui Q., Mi Y. (2020). Preparation of degradable magnetic temperature-and redox-responsive polymeric/Fe3O4 nanocomposite nanogels in inverse miniemulsions for loading and release of 5-fluorouracil. Colloid Surf. A..

[308] Trubiani O., Marconi G.D., Pierdomenico S.D., Piattelli A., Diomede F., Pizzicannella J. (2019). Human Oral Stem Cells, Biomaterials and Extracellular Vesicles: A Promising Tool in Bone Tissue Repair. Int. J. Mol. Sci..

[309] Moroni L., Schrooten J., Truckenmüller R., Rouwkema J., Sohier J., van Blitterswijk C.A. (2014). Tissue Engineering: An Introduction. Tissue Engineering.

[310] Stratton S., Shelke N.B., Hoshino K., Rudraiah S., Kumbar S.G. (2016). Bioactive polymeric scaffolds for tissue engineering. Bioact. Mater..

[311] Bardakova K.N., Akopova T.A., Kurkov A.V., Goncharuk G.P., Butnaru D.V., Burdukovskii V.F., Antoshin A.A., Farion I.A., Zharikova T.M., Shekhter A.B. (2019). From Aggregates to Porous Three-Dimensional Scaffolds through a Mechanochemical Approach to Design Photosensitive Chitosan Derivatives. Mar. Drugs.

[312] Nady N., Kandil S.H. (2018). Novel Blend for Producing Porous Chitosan-Based Films Suitable for Biomedical Applications. Membranes.

[313] Ambre A.H., Katti D.R., Katti K.S. (2013). Nanoclays mediate stem cell differentiation and mineralized ECM formation on biopolymer scaffolds. J. Biomed. Mater. Research. Part A.

[314] Jing X., Mi H.Y., Turng L.S. (2017). Comparison between PCL/hydroxyapatite (HA) and PCL/halloysite nanotube (HNT) composite scaffolds prepared by co-extrusion and gas foaming. Mater. Sci. Eng. C.

[315] Liu M., Zhang Y., Wu C., Xiong S., Zhou C. (2012). Chitosan/halloysite nanotubes bionanocomposites: Structure, mechanical properties and biocompatibility. Int. J. Biol. Macromol..

[316] Seda Tigli R., Karakecili A., Gumusderelioglu M. (2007). In vitro characterization of chitosan scaffolds: Influence of composition and deacetylation degree. J. Mater. Sci. Mater. Med..

[317] Das S.S., Neelam, Hussain K., Singh S., Hussain A., Faruk A., Tebyetekerwa M. (2019). Laponite-based Nanomaterials for Biomedical Applications: A Review. Curr. Pharm. Des..

[318] O’Brien F.J. (2011). Biomaterials & scaffolds for tissue engineering. Mater. Today.

[319] Das S.S., Hussain A., Verma P.R.P., Imam S.S., Altamimi M.A., Alshehri S., Singh S.K. (2020). Recent advances in liposomal drug delivery system of Quercetin for cancer targeting: A mechanistic approach. Curr. Drug Deliv..

[320] Harshita, Barkat M.A., Das S.S., Pottoo F.H., Beg S., Rahman Z. (2020). Lipid-Based Nanosystem As Intelligent Carriers for Versatile Drug Delivery Applications. Curr. Pharm. Des..

[321] Palacio H., Otalvaro F., Giraldo L.F., Ponchel G., Segura-Sanchez F. (2017). Chitosan-Acrylic Polymeric Nanoparticles with Dynamic Covalent Bonds. Synthesis and Stimuli Behavior. Chem. Pharm. Bull..

[322] Li Y., Lin J., Zhi X., Li P., Jiang X., Yuan J. (2018). Triple stimuli-responsive keratin nanoparticles as carriers for drug and potential nitric oxide release. Mater. Sci. Eng. C.

[323] Liu L., Gao X., Li X., Zhu G., Li N., Shi X., Wang Y. (2020). Calcium alendronate-coated composite scaffolds promote osteogenesis of ADSCs via integrin and FAK/ERK signalling pathways. J. Mater. Chem. B.

[324] Lowenberg C., Julich-Gruner K.K., Neffe A.T., Behl M., Lendlein A. (2020). Salt-Induced Shape-Memory Effect in Gelatin-Based Hydrogels. Biomacromolecules.

[325] Patel K.D., Kim T.H., Mandakhbayar N., Singh R.K., Jang J.H., Lee J.H., Kim H.W. (2020). Coating biopolymer nanofibers with carbon nanotubes accelerates tissue healing and bone regeneration through orchestrated cell- and tissue-regulatory responses. Acta Biomater..

[326] Li Y., Zhi X., Lin J., You X., Yuan J. (2017). Preparation and characterization of DOX loaded keratin nanoparticles for pH/GSH dual responsive release. Mater. Sci. Eng. C.

[327] Rebelo A.L., Bizeau J., Russo L., Pandit A. (2020). Carbohydrate-functionalized Collagen Hydrogels Modulate the Glycoenviroment of a Neuronal Primary Culture. Biomacromolecules.

